# A sensitive liquid chromatography–tandem mass spectrometry analytical method of steroid hormones in small blubber samples from four whale species

**DOI:** 10.1093/conphys/coag023

**Published:** 2026-04-13

**Authors:** Daniela Dulgheriu, Clare Andvik, Eve Jourdain, Katrine Borgå, Anders Ruus, Tore Haug, Richard Karoliussen, Jan Ludvig Lyche, Tor Einar Horsberg

**Affiliations:** Department of Paraclinical Sciences, Norwegian University of Life Sciences, Elizabeth Stephansens vei 15, 1433 Ås, Norway; Department of Biosciences, University of Oslo, Kristine Bonnevies hus, Blindernveien 31, 0316 Oslo, Norway; Department of Biosciences, University of Oslo, Kristine Bonnevies hus, Blindernveien 31, 0316 Oslo, Norway; Norwegian Orca Survey, Breivikveien 10, 8480 Andenes, Norway; Department of Biosciences, University of Oslo, Kristine Bonnevies hus, Blindernveien 31, 0316 Oslo, Norway; Department of Biosciences, University of Oslo, Kristine Bonnevies hus, Blindernveien 31, 0316 Oslo, Norway; Norwegian Institute for Water Research, Økernveien 94, 0579 Oslo, Norway; Institute of Marine Research, Fram Centre, Hjalmar Johansens gate 14, , 9007 Tromsø, Norway; Norwegian Orca Survey, Breivikveien 10, 8480 Andenes, Norway; Department of Paraclinical Sciences, Norwegian University of Life Sciences, Elizabeth Stephansens vei 15, 1433 Ås, Norway; Department of Paraclinical Sciences, Norwegian University of Life Sciences, Elizabeth Stephansens vei 15, 1433 Ås, Norway

**Keywords:** Baleen whale, cetacean, health, LC–MS/MS, marine mammals, reproduction, stranded whales, stress, toothed whale, validation

## Abstract

Steroid hormones can give an indication of stress, physiological health and reproductive status in whales; however, analysis is often limited by sample mass. We developed a sensitive analytical method using solid phase extraction–liquid chromatography–electrospray ionization–tandem mass spectrometry (LC–ESI–MS/MS) for the identification and quantification of eight steroid hormones (cortisol, cortisone, testosterone, androstenedione, progesterone, 11-deoxycortisol, 11-deoxycorticosterone and 17-hydroxyprogesterone) in small amounts of whale blubber (≤50 mg). Blubber biopsies were collected from stranded killer whales (*Orcinus orca; n* = 7), harbour porpoises (*Phocoena phocoena*; *n* = 3) and sperm whales (*Physeter macrocephalus*; *n* = 4), harvested common minke whales (*n* = 10; *Balaenoptera acutorostrata*) and free-living killer whales (*n* = 2) in Norway and the Barents Sea. Steroid hormones were separated using pentafluorophenyl propyl stationary phase and an optimized gradient programme with a total 20-min run. All samples (*n* = 26) were analysed at 50-mg sample mass, and a subset (*n* = 11) at 25 mg or lower. Low limit of detection and limit of quantification values resulted in high detection rates of hormones, and we found similar hormone concentrations between parallel samples and at the lowest tested sample mass (14 mg). Cortisol concentrations in sperm whales (4.3 ± 1.6 ng/g) were four times higher than stranded killer whales (1.1 ± 0.96 ng/g), which in turn were up to four times higher than free-living killer whales (0.23 ± 0.12 ng/g), harbour porpoise (0.64 ± 0.38 ng/g) and harvested minke whales (0.22 ± 0.13), possibly due to fatality type and duration of stress prior to death. We provide the first quantification of blubber steroid hormones in killer whale, minke whale, sperm whale and harbour porpoise, and in less than 50-mg sample mass. By minimizing sample mass without compromising precision or sensitivity, our method allows for steroid hormone analysis even on samples with limited availability, strengthening our ability to monitor and conserve marine mammal populations.

## Graphical Abstract

**Graphical Abstract:**
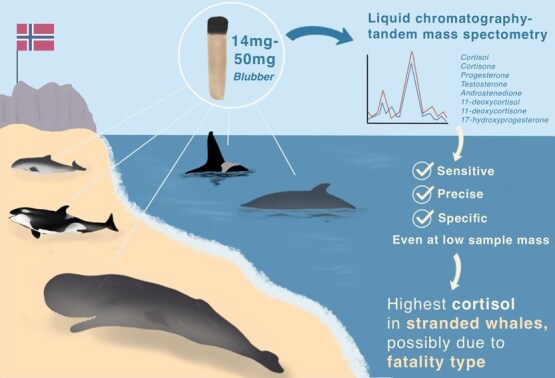

## Introduction

Steroid hormones provide insights into the health and fitness of marine mammals at both the individual and population levels, by providing a signal of stress, physiological health and reproductive status (Keay *et al.,* 2006; Kershaw and Hall, 2016; McCormick and Michael Romero, 2017). Corticosteroids, such as cortisol and its precursors cortisone and 11-deoxycortisol, and 11-deoxycorticosterone, a precursor to aldosterone, can give an indication of acute and chronic stress, as well as metabolic regulation, due to their role in lipid metabolism and gluconeogenesis (Peckett *et al.,* 2011; Kershaw and Hall, 2016). Reproductive steroid hormones, such as progesterone, 17-hydroxyprogesterone, testosterone and testosterone’s precursor androstenedione, can signal sexual maturity and pregnancy status (Luche *et al.,* 2020; Boggs *et al.,* 2019a). Steroid hormone data are thus instrumental for conservation efforts, for ecological studies and to assess effects of human activities (Wikelski and Cooke, 2006; Parsons *et al.,* 2015; Pallin *et al.,* 2022).

A range of biological matrices have been utilized for steroid hormone analysis in marine mammals, such as plasma (Ortiz and Worthy, 2000), serum (Legacki *et al.,* 2020), milk (West *et al.,* 2000), urine (Walker *et al.,* 1988) and even wax earplug (Trumble *et al.*, 2013). Such matrices are, however, reliant on the animal being either dead, small enough for live capture, or under managed care. Blubber, as an endocrinologically relevant and metabolically active tissue (Deslypere *et al.,* 1985), has become a widely used sample matrix due to the possibility of obtaining samples from not only dead animals, but also free-living individuals via remote biopsy sampling (Noren and Mocklin, 2012; Kellar *et al.,* 2015; Boggs *et al.,* 2019a).

Two main analytical techniques are utilized to measure steroid hormones in marine mammal blubber: enzyme immunoassays and liquid chromatography–tandem mass spectrometry (LC–MS/MS). Immunoassays are widely employed due to their relatively low cost and ease of application; however, they often suffer from cross-reactivity and limited specificity for various hormones (Hansen *et al.,* 2011; Hayden *et al.,* 2017). In addition, they usually require at least 100 mg of blubber per hormone for analysis, meaning that an analysis of just three or four hormones could require an entire biopsy sample (Mingramm *et al.,* 2020; Graham *et al.,* 2021; Pallin *et al.,* 2022). Conversely, LC–MS/MS offers comparable sensitivity, greater specificity and an ability to measure multiple hormones simultaneously, which enhances an understanding of hormonal interactions and allows for analysis in smaller samples (Boggs *et al.,* 2017; Wittmaack *et al.,* 2022). Sample masses for LC–MS/MS are commonly about 400 mg (Boggs *et al.,* 2017), with recent modifications reducing sample mass to 50 mg in humpback whales (*Megaptera novaeangliae*) for a simultaneous analysis of 10 steroid hormones (Wittmaack *et al.,* 2022). As obtaining blubber samples from whales can be challenging, especially in free-living animals, the already small samples are often divided for multiple analyses. Thus, investigating the minimum sample mass required for robust, accurate and reliable measurements of steroid hormones is essential to allow for simultaneous investigations of diet, chemical contaminants, genetics, health, etc. in the same individual, thereby providing knowledge for improved population monitoring and conservation strategies.

Due to species-specific differences in blubber composition, it is recommended that LC–MS/MS analyses be validated for each marine mammal species (Wittmaack *et al.,* 2022). Furthermore, establishing baseline hormone values for each species and tissue type is important for further studies of wild populations (Rolland *et al.,* 2012; Wasser *et al.,* 2017). To date, no studies have been published on blubber hormone concentrations in any population of killer whale (*Orcinus orca)*, harbour porpoise (*Phocoena phocoena)* or sperm whale (*Physeter macrocephalus*). Only progesterone concentrations in blubber of common minke whale (*Balaenoptera acutorostrata*, hereafter minke whale) from the Barents Sea have been quantified, using an immunoassay method (Mansour *et al.,* 2002).

In the present study, our objectives were to (1) validate a new LC–MS/MS analytical method of eight steroid hormones (cortisol, cortisone, 11-deoxycortisol, 11-deoxycorticosterone, progesterone, 17-hydroxyprogesterone, testosterone and androstenedione) in blubber of killer whales, sperm whales, harbour porpoise and minke whales, and (2) investigate the minimum required blubber mass for reliable and specific analysis. We further compare our results against what is known in the literature on how steroid hormone concentrations vary across fatality type, age and sex of marine mammals, as another means to validate our results.

## Materials and Methods

### Sampling

Blubber samples from harbour porpoise and sperm whale (*n* = 3 and *n* = 4, respectively) were collected from dead individuals found stranded along the Norwegian coast in 2020. Blubber samples from killer whales were collected from both stranded individuals from the Norwegian coast 2016–2021 (*n* = 7) and free-living individuals (*n* = 2) by remote biopsy sampling in 2021 in northern Norway. Blubber samples from minke whale (*n* = 10) were collected from individuals taken in the 2019 annual commercial harvest in the Barents Sea. A map showing the locations of sample collection can be found in Supplementary Fig. S1. Sample collection of stranded marine mammals was coordinated by the Norwegian Orca Survey as part of the stranded whales project at the University of Oslo, and of minke whales by the Norwegian Institute of Marine Research. Sampling methodology for the stranded and harvested individuals, including classifications of age (sub-adult/adult) and sex (female/male), is described in Andvik *et al.* (2021, 2023, 2024). Age and sex were unknown for the three harbour porpoises. The two free-living killer whales were classified as adult male due to secondary sex characteristics (body size and dorsal fin length and shape; Bigg, 1982). The decomposition state of each stranded individual was coded based on established protocols where 2 = freshly deceased and no bloating, 3 = moderately decomposed with mild to moderate bloating and 4 = advanced decomposition with major bloating/organs beyond recognition (Kuiken and García Hartmann, 1991). Biopsy sampling followed the protocols described in Jourdain *et al.* (2020) and was conducted under permit FOTS-ID: no. 24249, report no. 20/151683. Samples were frozen after collection and until analysis (−20°C for stranded whales and minke whales, and −80°C for free-living killer whales). There is evidence that sample degradation and storage temperature may affect hormone concentrations in whale blubber (Trana *et al.,* 2016); however, progesterone concentrations remained unchanged in samples stored at −20°C over 17 years (Pallin *et al.,* 2018). All stranded whales had decomposition codes of either 2 or 3, and we assume in the present study that any effect of sample degradation and storage on hormone concentrations is minimal.

### Sub-sampling and homogenization

For the harbour porpoises, minke, sperm and stranded killer whales (*n* = 24), a large piece of full depth blubber (approximately 50 g) was homogenized using a Knife Mill Grindomix GM 200 machine (RETSCH, Haan, Germany) and dry ice. Traces of associated skin were homogenized with the blubber, with the amount varying by species and individual. Replicates of 500-mg pooled samples were used for a sample preparation comparison study to evaluate the steroid extraction efficiency, and after optimizing sample preparation, the sample mass was scaled down to 50 mg.

Blubber mass from the free-living killer whales (*n* = 2) was limited; thus, to avoid sample loss in the Knife Mill Grindomix, approximately 100 mg of blubber was ground on liquid nitrogen using an agate pestle and mortar until a powder-like consistency was achieved. Using an analytical balance, approximately 50 mg was weighed into 2-ml GK 60 Precellys lysing tubes (Bertin Technologies, Montigny-le-Bretonneux, France) and kept at −80°C. We checked that this alternative homogenization method did not result in differences in hormone quantification by measuring steroid hormones in 50- and 25-mg samples from one killer whale (ID Oo3) homogenized using both methods and found no significant difference (see Supplementary Table S1).

To investigate the possible effect of sample mass on hormone concentrations, different sample masses from four individuals were analysed after homogenization by pestle and mortar. These consisted of stranded killer whale Oo3 (50 and 26 mg); free-living killer whales 116 and 113 (~50 and ~25 mg) and stranded sperm whale SW7 (five masses: 53, 38, 31, 19 and 14 mg). The initial homogenization of sperm whale SW7 included skin; however, when this individual was selected to assess the effect of sample mass, a separate batch was homogenized and only blubber used, hence the differences in steroid hormone concentrations for this individual across the two assessments.

### Chemical and reagents

Cortisol, cortisone, testosterone, androstenedione, progesterone, 11-deoxycortisol, 11-deoxycorticosterone and 17-hydroxyprogesterone and their respective corresponding stable isotope-labelled standards (cortisol-d_4_, cortisone-d_8_, ^13^C_3_-testosterone, ^13^C_3_-androstenedione, progesterone-d_9_, 11-deoxycortisol-d_5_, 11-deoxycorticosterone-d_7_ and 17-hydroxyprogesterone-d_8_) were of analytical or certified reference material grade and provided from Merck-Sigma (Darmstadt, Germany). Ultrapure water (18.2 Ω, TOC <1) was produced by the Elga Ultrapure Laboratory water purification system (Lane End, UK). The solvents and reagents (acetonitrile, methanol, formic acid, hexane) were high-performance liquid chromatography (HPLC), LC–MS or analytical grade and supplied from VWR International (Radnor, USA).

Individual stock solutions of 1 mg/ml of each steroid were prepared by dissolving the powder of each compound in methanol. Solutions were stored at −20°C. Working solutions were prepared by dilution with methanol. The calibration standards were prepared in 50% methanol/water (v/v).

The internal standard (IS) mixture working solution used to spike each sample contained the following: 10 ng/ml of cortisol-d_4_, cortisone-d_8_, ^13^C_3_-testosterone, ^13^C_3_-androstenedione, progesterone-d_9_, 11-deoxycortisol-d_5_, 11-deoxycorticosterone-d_7_ and 17-hydroxyprogesterone-d_8_. It was prepared in methanol and stored at −80°C until subsequent analysis.

### Steroid hormone extraction

Homogenized samples were thawed on ice, and 25-μl isotope-labelled internal standard working solution prepared in methanol was added to each 50-mg blubber sample. The volume of the added internal standard solution was accordingly adjusted to the measured sample mass. An isotope dilution technique was used to correct for variations during sample preparation and instrumental response, ensuring a high accuracy of the quantification process. The isotope dilution principle allows for the accurate determination of the analyte concentration by comparing the isotopic ratio of the labelled and unlabelled form (Humston *et al.,* 2010).

A volume of 1400 μl of 90% acetonitrile/methanol (v/v) with 0.2% formic acid was added to each 2-ml GK60 Precellys tube containing the homogenized sample. Each 2-ml reinforced tube contains one ceramic bead of 6.0- and 0.7-mm garnet flakes and is especially designed for hard tissue grinding, ensuring a more uniform homogenate and better extraction efficiency compared to other bead materials tested (data not shown). Samples were then further homogenized for three cycles of 20 s at 6800RPM speed (hard homogenization programme) on a Precellys 24 bead homogenizer (Bertin Technologies, Montigny-le-Bretonneux, France) and centrifuged at 2400*g* for 5 min using an Heraeus Pico 21 centrifuge (Thermo Fisher Scientific, Waltham, USA). The supernatant was transferred to a new 15-ml polypropylene tube (WVR, Radnor, USA), and one more extraction was carried out, followed by centrifugation as described above. Approximately 2700 μl of supernatant, as a result of two homogenizations, were then cleaned thrice with 2750-μl hexane to remove the fat content, as recommended in Wittmaack *et al.* (2022). Samples were vortexed for 30 s, then centrifuged for 3 min at 2850*g* using an Allegra X-12R centrifuge (Beckman Coulter, California, USA), and the hexane layer was discarded.

Extracts were then incubated at −20°C overnight to increase the analyte yield. The next day, 2500 μl of the sample extract were passed through 3-ml (60 mg) Oasis PRiME hydrophilic–lipophilic balance (HLB) (Waters, Milford, USA) reverse-phase polymeric sorbent solid phase extraction (SPE) cartridges. Sample extracts were applied straight to the cartridge and allowed to drop down slowly, then vacuum dried for 20 s. No wash step was performed. The Oasis PRiME HLB cartridges allow simplified workflow, which mean no conditioning or equilibration is needed, removing greater than 95% of matrix interferences. This step helped to mitigate the difficulties of removing fat from the samples whilst retaining the compound of interest, and in obtaining cleaner extracts with minimal matrix effects (MEs) and good recoveries. Sample extracts were then evaporated until dry under a gentle nitrogen stream at 37°C by using a Reacti-Vap Evaporator (Thermo Fisher Scientific, Waltham, USA). The residue was reconstituted in 100 μl of 50% methanol/water (v/v), filtered with Spin-X centrifuge tube filter, 0.22 μm (Costar, Washington, DC, USA), and transferred to an HPLC vial with insert (Agilent, Santa Clara, USA).

### Liquid chromatography and mass spectrometry

Sample extracts (20 μl) were injected into an LC–MS/MS system consisting of an Agilent 1100 binary pump, degasser and autosampler with thermostat (Agilent Technologies, Santa Clara, USA) coupled to a Sciex API 4000 triple–quadrupole mass spectrometer, equipped with electrospray ionization interface (SCIEX, Toronto, Canada). Chromatographic separation was performed using a Phenomenex Kinetex-F5 column, 100 × 2.1 mm, 2.5-μm core-shell particles (Phenomenex, Torrance, USA). The mobile phase consisted of water and 0.1% formic acid (A) and acetonitrile with 0.1% formic acid (B). The flow rate was 250 μl/min, and the column temperature was kept at 35°C. The reverse-phase LC gradient profile was optimized as follows: 15% B to 85% B for 7.4 min, 90% at 10 min, 95% at 10.5 min, 98% at 12 min, 100% at 12.2 min, 15% at 15.1 min, stop time at 20 min. The autosampler temperature was set at 5°C. A divert valve was used, and the HPLC flow was directed to the mass spectrometer from 7 to 13 min.

The separated compounds were detected in negative and positive ionization—multiple reaction monitoring (MRM) mode in the same run analysis, selecting one precursor ion to two product ion transitions (a quantifier and a qualifier) for each compound (Table 1). The quantifier ion (usually the ion with the highest signal) is used to quantify the analyte, while the qualifier ion is used to identify the analyte. The ion ratio of the quantifier and qualifier gives evidence about the correct identity of the steroids being monitored. Instrument parameter optimization for each analyte was achieved by infusing standard solutions of 200 ng/ml steroid hormone prepared in 50% methanol/water (v/v) with 0.1% formic acid, direct into the Turbo Ion Spray source, through an infusion pump with 1-ml syringe (Hamilton, Reno, USA) set at 20 μl/min flow rate.

**Table 1:** The optimized mass spectrometer parameters in eight steroid hormones and eight corresponding isotope-labelled internal standards with their respective precursor/product ion pairs and their respective mass to charge ratio (m/z) values in MRM acquisition mode

**Compound**	**Ion mode**	**RT (min)**	**Precursor ion (m/z)**	**DP(V)**	**Quantifier ion (product ion 1)**			**Qualifier ion (product ion 2)**		
					**m/z**	**CE(V)**	**CXP(V)**	**m/z**	**CE(V)**	**CXP(V)**
Cortisol	ESI neg	9.01	407.4	−66	331	−23	−7	297	−45	−5
Cortisol-d_4_	ESI neg	9.01	411.3	−65	335	−26	−7	128	−54	−6
Cortisone	ESI neg	9.26	405.5	−60	329	−19	−7	301	−29	−7
Cortisone-d_8_	ESI neg	9.26	411.4	−60	337	−22	−6	309	−30	−8
Androstenedione	ESI pos	11.15	287.3	90	97	35	4	109	35	5
^13^C-Androstenedione	ESI pos	11.15	290.2	84	100	33	5	112	33	6
Testosterone	ESI pos	10.73	289.3	83	97	32	4	109	36	5
^13^C_3_-Testosterone	ESI pos	10.73	292.2	90	100	33	6	112	34	6
11-Deoxycorticosterone	ESI pos	10.96	331.3	85	97	33	5	109	36	8
11-Deoxycorticosterone-d_7_	ESI pos	10.96	338.4	96	100	36	5	113	37	6
17-Hydroxyprogesterone	ESI pos	11.23	331.4	85	97	35	4	109	41	5
17-Hydroxyprogesterone-d_8_	ESI pos	11.23	339.3	91	100	35	5	113	37	6
Progesterone	ESI pos	12.06	315.3	86	109	37	5	97	33	4
Progesterone-d_9_	ESI pos	12.06	324.4	87	100	34	6	113	33	6
11-Deoxycortisol	ESI pos	10.16	347.3	80	97	37	5	109	40	5
11-Deoxycortisol-d_5_	ESI pos	10.16	352.3	87	100	41	5	113	42	6

Retention time (RT) DP (decluster potentials), CE (collision energies) and CXP (collision exit potentials) specific to each steroid analyte.

Six steroid hormones (testosterone, androstenedione, progesterone, 11-deoxycorticosterone, 17-hydroxyprogesterone and 11-deoxycortisol) were analysed in positive MRM mode using [M + H]^+^ as precursor ions, while cortisol and cortisone were analysed in negative ion MRM mode using [M + HCOO]^−^ as precursor ions in the same run divided in two MS scan periods for a total analysis time of 20 min (Table 1). Cortisol and cortisone can form very abundant and stable formate adducts with formic acid added to the mobile phase in negative electrospray ionization mode. Its sensitivity is enhanced by using the [M + HCOO]^−^ adducts in negative MRM mode, at least 3× compared to positive MRM mode.

The software used for controlling this equipment, and acquiring and processing the data was Analyst Version 1.7 (SCIEX, Toronto, Canada). Manual integrations of peaks were used when necessary. Further method parameters are listed in Supplementary Text 1.

### Method validation

Validation was performed following the guidelines of Commission Implementing Regulation EU 2021/808 of 22 March 2021 on the performance of analytical methods and the interpretation of the results (2002/657/EC). Due to the endogenous nature of the steroid hormones, validation was conducted by spiking blubber homogenate with the corresponding isotope-labelled standard analogues of each compound as a surrogate standard. To avoid variation during the validation study, replicates of the same sample (SW4) were used. The following validation parameters were assessed: specificity, limit of detection (LOD), limit of quantification (LOQ), linearity, precision, accuracy, recovery and MEs.

#### Sensitivity and linearity

LOD was based on 3× signal-to-noise ratio (S/N), and LOQ was estimated as the lowest concentration point of the calibration curves used in the validation study. The noise signal was estimated as three times the matrix blanks (*n* = 3). The LOD was defined as the lowest concentration of each analyte that could be reliably differentiated from the background noise of a blank sample. The LOD and LOQ for each steroid hormone are presented in Table 2. The calibration standards were prepared in a dilution solution (50% methanol/water v/v) as a surrogate matrix based on the correction factors calculated for each compound related to their respective total recovery values, which included both MEs and extraction efficiencies. The correction factors were calculated by dividing the mean of the analyte peak area from six replicates (50 mg blubber) spiked at 5 ng/g with isotope-labelled standard equally in concentration with corresponding un-labelled steroids, to the analyte peak area of a neat isotope-labelled standard in 50% MeOH/H_2_O v/v solution at the same concentration. The calculated correction factors were applied for both un-labelled and labelled steroid hormones. The highest calibrator containing a mixture of eight steroid hormones was prepared in dilution solution and further diluted to obtain a seven-point calibration curve with the LOQ as the lowest calibrator in the standard curve. The same amount of the internal standard mixture corresponding to 5 ng/g concentration was added to each calibrator. A linear regression was used for the quantitative assay.

**Table 2:** Steroid hormone concentrations (ng/g) in 50 mg of blubber from whales of differing species, age, sex and blubber lipid percentage

**Species**	**Age and sex**	**Whale ID**	**Decomposition code**	**Cortisol (ng/g)**	**Cortisone (ng/g)**	**Androstenedione (ng/g)**	**Testosterone (ng/g)**	**Progesterone (ng/g)**	**11-Deoxycorticosterone (ng/g)**	**17-Hydroxyprogesterone (ng/g)**	**11-Deoxycortisol (ng/g)**
Minke whale (harvested)	Adult female (pregnant)	Kato 67	NA	0.17	0.28	3.3	0.43	20	<0.20	<0.30	<0.20
		Kato 68	NA	0.16	0.29	1.0	<0.20	60	<0.20	<0.30	<0.20
		Kato 70	NA	0.20	0.52	1.8	0.20	100	<0.20	<0.30	<0.20
		Kato 78	NA	0.29	0.30	2.4	0.28	63	<0.20	<0.30	<0.20
	Adult male	Kato 69	NA	0.19	0.35	2.3	0.25	0.97	<0.20	<0.30	<0.20
		Kato 71	NA	0.20	0.54	4.6	1.0	0.63	<0.20	<0.30	<0.20
		Kato 72	NA	0.12	0.42	2.5	2.5	2.2	<0.20	<0.30	<0.20
		Kato 73	NA	0.21	0.29	4.4	0.75	1.3	<0.20	<0.30	<0.20
		Kato 75	NA	0.12	1.4	23	1.3	5.3	<0.20	<0.30	<0.20
	Sub-adult female	Kato 74	NA	0.55	0.35	12	1.2	0.55	<0.20	<0.30	<0.20
Killer whale (free-living)	Adult male	116	NA	0.15	0.18	1.5	4.6	0.72	<0.20	<0.30	<0.20
		113	NA	0.31	0.16	1.0	2.0	<0.20	<0.20	<0.30	<0.20
Killer whale (stranded)	Adult female	Oo2	3	2.5	1.7	1.0	<0.20	2.9	<0.20	<0.30	1.1
		Oo3	3	2.0	1.1	4.0	0.22	68	1.6	<0.30	1.6
		Oo7	3	0.72	1.2	2.2	0.83	3.5	<0.20	<0.30	1.6
	Adult male	Oo4	3	0.22	0.27	1.4	0.30	<0.20	<0.20	<0.30	<0.20
		Oo8	2	1.2	1.0	1.1	1.8	1.2	<0.20	<0.30	2.2
	Sub-adult male	Oo5	2	0.11	0.36	2.9	<0.20	0.27	<0.20	<0.30	<0.20
	Sub-adult female	Oo11 “Elida”	2	29	4.1	0.87	2.5	0.87	0.78	1.2	4.8

(Continued)

**Table 2:** Continued

**Species**	**Age and sex**	**Whale ID**	**Decomposition code**	**Cortisol (ng/g)**	**Cortisone (ng/g)**	**Androstenedione (ng/g)**	**Testosterone (ng/g)**	**Progesterone (ng/g)**	**11-Deoxycorticosterone (ng/g)**	**17-Hydroxyprogesterone (ng/g)**	**11-Deoxycortisol (ng/g)**
Harbour porpoise (stranded)	Unknown	HP3	2	0.25	0.42	1.6	0.46	0.28	0.63	<0.30	<0.20
		HP4	2	0.64	0.71	6.0	0.66	<0.20	<0.20	<0.30	1.7
		HP5	2	1.0	0.72	3.4	0.62	<0.20	<0.20	<0.30	0.65
Sperm whale (stranded)	Adult female	SW7	3	2.9	1.1	2.3	0.79	1.5	1.1	<0.30	0.83
	Adult male	SW2	2	3.2	1.2	2.6	1.8	<0.20	<0.20	<0.30	1.2
		SW3	2	4.7	2.6	5.5	0.84	<0.20	<0.20	<0.30	2.5
	Sub-adult male	SW4	3	6.3	1.8	3.1	1.6	0.67	<0.20	<0.30	0.79
LOQ:				0.080	0.060	0.50	0.50	0.50	0.50	0.60	0.50
LOD:				0.030	0.020	0.20	0.20	0.20	0.20	0.30	0.20
% >LOQ				100	100	100	58	69	4.0	15	42
% >LOD				100	100	100	88	77	4.0	15	42

LOD and LOQ are indicated for each of the eight steroid hormones, as well as percentage of samples found over the LOD and LOQ.

The response of each analyte is linear with correlation coefficients >0.99 (*r*). The hormone concentration in each sample was calculated by an internal standard method using the peak area ratio (area of the analyte divided by the area of the corresponding isotope-labelled internal standard) and linear regression analysis, with a 1/*x* weighting. Concentrations were expressed in nanograms of hormone per gram of blubber tissue (ng/g).

#### Quality assurance

The quality control (QC) data provide an estimate of intermediate precision and repeatability. The precision of the analytical method was determined by evaluating the closeness of a set of analytical results obtained from a series of replicate samples and was expressed in terms of the relative standard deviation (RSD%) for those measurements.

Intra-assay precision and accuracy for this method were calculated using six replicates of the QC blubber samples spiked at the same concentration with the corresponding isotope-labelled steroids (5 ng/g) analysed during a single analytical run, in the validation study. We verify that the isotope-labelled standard produces a mass spectrum (MS) signal intensity identical to that of the corresponding standard (pure steroid target analyte).

Inter-assay precision and accuracy were calculated by spiking a blubber sample with a known concentration of unlabelled steroid standards and isotope-labelled internal standards (to provide three in-house QC samples) and were used for each run. Replicates of sample OO4 killer whale as “blank matrix” were used for this purpose.

Precision (should be less than 15%) was expressed as the RSD% of the control samples spiked at the same concentration. Accuracy was expressed as the ratio of calculated concentration to known concentration (100 ± 20%). QC samples were fortified with internal standard (5 ng/g) and pure steroid hormones standards at a concentration range of 0, 0.5, 1.0 and 4.0 ng/g. Intra-assay accuracy ranged from 96.1% to 102.5%, and RSD% from 2.18% to 8.29% (Supplementary Table S2). Inter-assay accuracy ranged from 92.6% to 111.8%, and RSD% from 1.29% to 11.0% (Supplementary Table S3). The ideal sample for inter-assay QC purpose would be “Elida” killer whale blubber replicates, which contains almost all the analysed endogenous steroids. Unfortunately, due to the limited amounts of that specific sample, we chose to use replicates of the aforementioned blubber sample spiked at low–medium–high concentrations with very good inter-day reproducibility.

#### MEs and recovery

Matrix effects (MEs) refer to how the measurement of the target steroid may be affected by interference with endogenous or exogenous compounds present in the sample or sample preparation consumables. The matrix components interfere with the ionization of the analyte causing ion suppression (suppressed analyte signal) (<100%) or enhancement (enhanced analyte signal, >100%). MEs were evaluated by comparing the peak area of each analyte added to a dilution solution (50% MeOH/H_2_O v/v) of a neat standard (without the matrix) with the peak area for the same concentration of analytes post-spiked to the extracted “blank” sample. Blubber sample extract was spiked with an isotopically labelled steroid standard (5 ng/g concentration) after sample preparation (post-spiked) right before LC–MS/MS analysis. ME for each steroid was calculated by using the ratio of the peak area of the analyte in the presence of the matrix to the peak area in its absence (a neat solution of the analyte in 50% MeOH/H_2_O v/v), and was expressed as percentages (Matuszewski *et al.,* 2003).


$$ \mathrm{ME}\ \left(\%\right)=\frac{\begin{array}{c}\mathrm{peak}\ \mathrm{area}\ \mathrm{postspiked}\ \mathrm{matrix}\ \mathrm{extract}\\ \mathrm{IS}-\mathrm{peak}\ \mathrm{area}\ \mathrm{matrix}\ \mathrm{extract}\end{array}}{\mathrm{peak}\ \mathrm{area}\ \mathrm{neat}\ \mathrm{standard}\ \mathrm{solution}\ \mathrm{IS}}\cdotp 100 $$

MEs ranged from 65% to 91% (Supplementary Table S4). We used a sample clean-up procedure and stable isotope-labelled internal standards (IS) to fully or partially correct for analyte losses during sample pre-treatment and matrix-related signal suppression or enhancement during analyte ionization, which could influence the accuracy and precision of the quantitative method.

Extraction recoveries (ERs) were calculated by comparing the peak areas of the matrix, spiked with a mixture of labelled isotopologues of the target analytes, before and after extraction. Six pre-spiked blubber samples were used for this experiment. Replicates of 50-mg blubber samples were spiked at 5 ng/g concentration and extracted as described above. The mean analyte peak area of these replicates (pre-spiked) is used for calculation. A “blank” blubber sample was spiked at the same concentration after sample processing (post-spiked).


$$ \mathrm{ER}\left(\%\right)=\frac{\begin{array}{c}\mathrm{peak}\ \mathrm{area}\ \mathrm{prespiked}\ \mathrm{matrix}\ \mathrm{extract}\ \mathrm{IS}\\- \ \mathrm{peak}\ \mathrm{area}\ \mathrm{matrix}\ \mathrm{extract}\end{array}}{\mathrm{peak}\ \mathrm{area}\ \mathrm{postspiked}\ \mathrm{matrix}\ \mathrm{extract}\ \mathrm{IS}}\cdotp 100 $$

The extraction recoveries of all hormones ranged from 88% to 117%, which are within acceptable ranges for sensitivity and precision (Supplementary Table S4).

The total recoveries (RTs) were calculated by comparing the mean peak area of six pre-spiked blubber matrix samples with the peak area of a neat standard at the same concentration.


$$ \mathrm{RT}\left(\%\right)=\frac{\begin{array}{c}\mathrm{peak}\ \mathrm{area}\ \mathrm{prespiked}\ \mathrm{matrix}\ \mathrm{extract}\ \mathrm{IS}\\ - \ \mathrm{peak}\ \mathrm{area}\ \mathrm{matrix}\ \mathrm{extract}\end{array}}{\mathrm{peak}\ \mathrm{area}\ \mathrm{neat}\ \mathrm{standard}\ \mathrm{solution}\ \mathrm{IS}}\cdotp 100 $$

The total recovery correction factors were used for building standard-based calibration curves (2.6).

Carryover was determined by injecting a solvent blank multiple times after the highest calibration standard. No carryover was observed in the solvent blanks. Each analytical run included both needle wash and solvent blanks to account for any background contamination.

### Data treatment

Data treatment and statistical analysis was conducted in R (v4.4.3: R Core Team, 2025). Values <LOD were replaced using distribution-based multiple imputation, using a *beta* distribution with α = 3 and β = 1 to retain the pattern of the dataset (Baccarelli *et al.,* 2005). The detection rate for the different hormones is presented in Table 2. Imputed values were used for calculating summary statistics for all hormones. In total, there were nine imputed values representing 6.2% of the total dataset.

The effect of sample mass was tested on a subset of samples (*n* = 11; see section 2.2) using a mixed linear effects model with hormone concentration as the response variable, sample mass as the fixed effect and whale ID as a random effect. Only cortisol, cortisone, progesterone, testosterone and androstenedione were tested due to low detection of 11-deoxycorticosterone, 17-hydroxyprogesterone and 11-deoxycortisol in these samples. Contrasts and confidence intervals were calculated using the package “emmeans” (Lenth, 2021) and back-transformed. When reporting results using log10-transformed hormone concentrations, the effect of an explanatory variable is interpreted as a multiplicative factor where if the point estimate and confidence intervals are close to 1, then the effect is minimal and not practically significant.

Due to low sample size and the presence of multiple confounding factors, statistical analyses were not conducted when comparing steroid hormone concentrations between species, age and sex. Summary statistics were instead presented and compared.

## Results

### Specificity and selectivity

**Figure 1:**
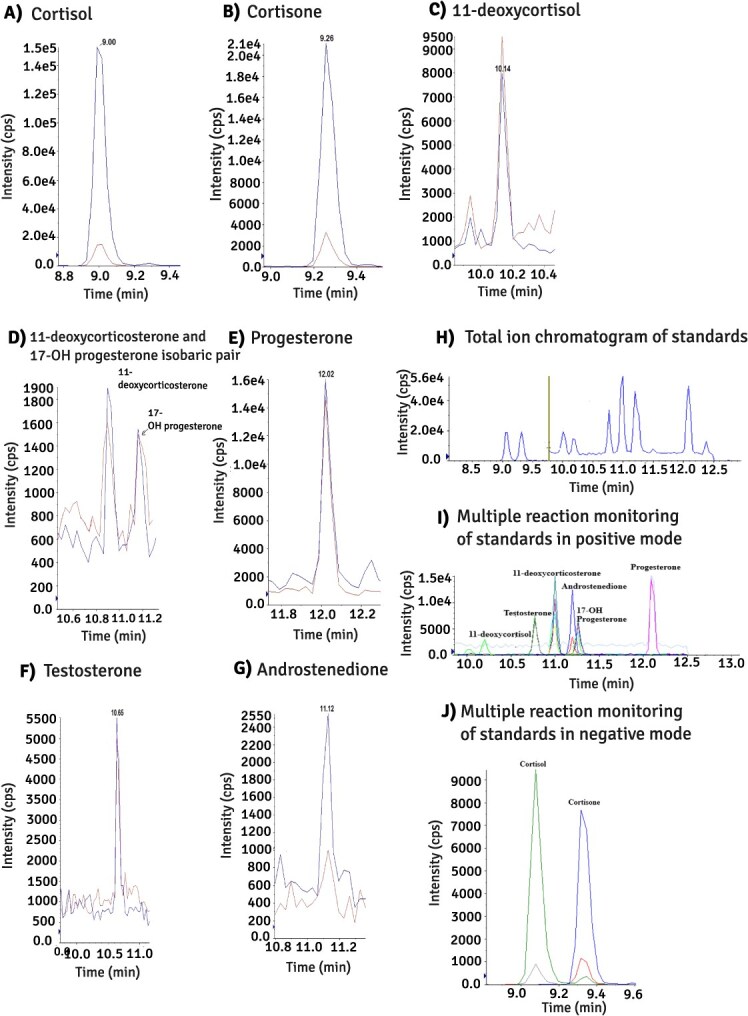
Extracted ion chromatograms (XIC) of endogenous steroid hormones (**A**) cortisol, (**B**) cortisone, (**C**) 11-deoxycortisol, (**D**) 11-deoxycorticosterone and 17-OH progesterone (isobaric steroids). (**E**) Progesterone, (**F**) testosterone and (**G**) androstenedione, measured in killer whale blubber. Testosterone was measured in adult male killer whale Oo8, and the others in sub-adult female killer whale Oo11 “Elida.” Blue/red chromatograms represent the quantifier/qualifier ion pairs for each measured steroid hormone, respectively. Steroid hormone standards (0.5 ng/ml) are presented as (**H**) the total ion chromatogram, (**I**) XIC of the MRM transitions in positive mode and (J) XIC of the MRM transitions in negative mode.

Specificity was checked for all analysed samples, and we detected no interfering peaks at the respective retention time of each steroid (Fig. 1). Isobaric steroids (identical masses but differing in structure) can contribute to errors even with the high specificity afforded by tandem mass spectrometry. The chromatographic separation using a pentafluorophenyl propyl core-shell column with small particles and multiple interactions, and the optimization of the gradient programme, contributed to a high resolution of peaks in terms of selectivity. Our method could successfully separate the 11-deoxycorticosterone (RT = 10.91 min) and 17-hydroxyprogesterone (RT = 11.23 min) isobaric pair (Fig. 1).

### Hormone detection in 50-mg samples

Cortisol, cortisone and androstenedione were quantified in 100% of the 50-mg samples (*n* = 26). Testosterone was quantified >LOQ in 15 of the 26 samples (58%), including all but two of the confirmed male individuals, with additional eight samples quantified between LOD and LOQ. Progesterone was quantified >LOQ in 18 of the 26 samples (69%), including all of the confirmed female individuals, with an additional two samples between the LOD and LOQ (Table 2). 11-Deoxycortisol was quantified >LOQ in 11 of the 26 samples (42%), with the other samples <LOD, which included all the minke whales and free-living killer whales. 11-Deoxycorticosterone was quantified >LOQ in four samples: one harbour porpoise, two stranded killer whales and the stranded female sperm whale. 17-Hydroxyprogesterone was quantified in just one sample, the female sub-adult killer whale Oo11 “Elida,” with the remaining 25 samples <LOD (Table 2).

### Reduction of sample mass

We tested the effect of sample mass on four individuals, comparing 50- and 25-mg sample mass. The predicted effect size of sample mass on the concentrations of all hormones was negligible, with back-transformed point estimates for all hormones very close to 1 (Table 3). The rate of detection was also the same regardless of the tested sample mass in our study (50 vs 25 mg or less), with samples that had hormone concentrations <LOD at the 50-mg sample mass also <LOD at lower sample masses, and there were no samples <LOD at lower sample masses that were detected at 50 mg (Table 3). We tested reducing the sample masses further in the blubber from a sperm whale (ID SW7) and quantified similar hormone concentrations even at the lowest tested sample mass of 14 mg (Table 3; Supplementary Fig. S2). The standard deviation and precision (measured as RSD%) were low for each species and steroid hormone, ranging from standard deviations of 0.0014 to 0.29 and RSD% of 0.98% to 9.2% (Table 3). Sensitivity remained good even with low sample mass (Supplementary Fig. S2).

**Table 3:** Blubber steroid hormone concentrations in four whale individuals: stranded killer whale Oo3, stranded sperm whale SW7 and free-living killer whales 116 and 113

**ID**	**Mass (mg)**	**Cortisol**			**Cortisone**			**Testosterone**			**Progesterone**			**Androstenedione**		
		ng/g	SD	RSD	ng/g	SD	RSD	ng/g	SD	RSD	ng/g	SD	RSD	ng/g	SD	RSD
Oo3	50	1.9	0.028	1.5	0.93	0.016	1.7	<0.5	N/A	N/A	68	0.28	0.41	4.0	0.085	2.9
Oo3	26	1.9			0.95			<0.5			68			4.1		
SW7	53	1.6	0.049	3.0	0.30	0.0030	1.0	0.69	0.013	1.9	<0.5	N/A	N/A	1.9	0.072	3.9
SW7	38	1.7			0.30			0.67			<0.5			2.0		
SW7	31	1.6			0.30			0.67			<0.5			1.9		
SW7	19	1.7			0.30			0.66			<0.5			1.8		
SW7	14	1.6			0.30			0.69			<0.5			1.8		
116	41	0.15	0.0014	0.98	0.18	0.057	3.1	4.6	0.050	1.1	0.72	0.011	1.5	1.5	0.057	3.9
116	24	0.14			0.19			4.5			0.70			1.4		
113	49	0.31	0.012	3.8	0.16	0.016	9.2	2.0	0.021	1.1	<0.2	N/A	N/A	1.0	0.028	2.7
113	28	0.33			0.18			2.0			<0.2			1.1		
**Mixed effect model estimates and coefficients**																
Estimate		0.99			1.0			1.0			1.0			1.0		
Standard error		1.0			1.0			1.0			1.0			1.0		
Confidence intervals		0.99–1.0			0.99–1.0			0.99–1.0			0.99–1.0			0.99–1.0		
*P* value		0.76			0.26			0.64			0.43			0.36		

All samples were homogenized using pestle and mortar and liquid nitrogen and were analysed at different masses. Concentrations are presented as nanograms per gram, and the SD and precision (RSD%) are presented. A mixed effect model on 11 samples from four individuals was fitted to investigate the effect of sample mass and had the following format: log10(hormone) ~ mass + (1 | ID), where ID was the ID of the individual whale. Back-transformed fixed effect estimates, standard error, confidence intervals and *P* values are presented. An estimate close to 1 indicates a negligible effect of weight on hormone concentrations.

### Concentrations in relationship to species, sex and age

**Figure 2:**
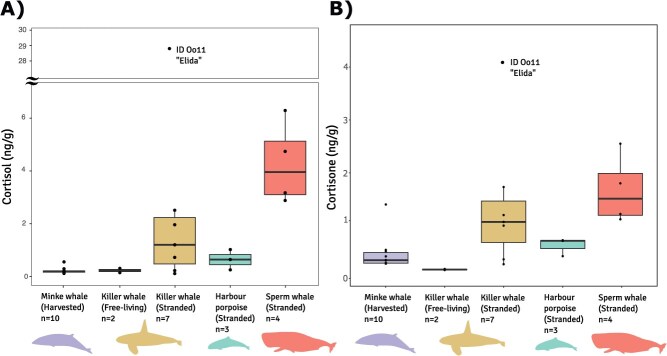
(**A**) Cortisol and (**B**) cortisone concentrations (ng/g) in four species of whale sampled from Norway, including both stranded and free-living killer whales. Note that the *y* axis is on different scales.

Cortisol and cortisone concentrations varied greatly between the species. Sperm whales had the highest mean cortisol concentrations (4.3 ± 1.5 ng/g), followed by stranded killer whales (1.1 ± 0.96 ng/g, not including ID Oo11 “Elida” with cortisol concentrations of 28.8 ng/g) and harbour porpoises (0.64 ± 0.38 ng/g; Table 2, Supplementary Table S5). Concentrations in the free-living killer whales and harvested minke whales were the lowest (0.23 ± 0.12 and 0.22 ± 0.13 ng/g, respectively) (Table 2, Fig. 2A, Supplementary Table S5). Cortisone concentrations followed a similar pattern, but with stranded killer whale and sperm whale concentrations similar, and approximately five times higher concentrations in stranded killer whales than free-living killer whales (Fig. 2B, Table 2, Supplementary Table S5). Within the minke whales, adult females (all of which were pregnant) had the highest progesterone concentrations (61 ± 34 ng/g), followed by 2.1 ± 1.9 ng/g for adult males and 0.55 ng/g for the sub-adult (non-pregnant) female (Table 2, Supplementary Table S5, Supplementary Fig. S3). Mean androstenedione concentrations were 2.1 ± 0.95 ng/g for adult females, 7.4 ± 8.9 ng/g for adult males and 12 ng/g for the sub-adult female (Table 2, Supplementary Table S5, Supplementary Fig. S5). Mean testosterone concentrations were 0.25 ± 0.13 ng/g for adult females, 1.2 ± 0.83 ng/g for adult males and 1.2 ng/g for the sub-adult female (Table 2, Supplementary Table S5). We found the highest testosterone levels in an adult male killer whale and a sub-adult male sperm whale (Table 2, Supplementary Fig. S4).

## Discussion

We have developed and validated an LC–MS/MS method for the reliable and accurate simultaneous quantification of eight steroid hormones (cortisol, cortisone, testosterone, androstenedione, progesterone, 11-deoxycortisol, 11-deoxycorticosterone and 17-hydroxyprogesterone) in 50 mg of whale blubber from four species, with indications that it can further be applied to samples with as little as 14-mg sample mass. We achieved low LOD and LOQs for all hormones, which resulted in high detection frequencies, especially for cortisol and cortisone (100% detection for each). Our LOD for cortisol was 0.03 ng/g, and 0.02 ng/g for cortisone, which is approximately 40 times lower LOD than a similar method quantifying steroid hormones in the same mass of whale blubber (50 mg) (Wittmaack *et al.,* 2022), and at least 10 times lower than other LC–MS/MS methods using greater sample masses (Boggs *et al.,* 2019b; 2017). The LODs were comparable to immunoassay techniques (Pallin *et al.,* 2022). LODs were, however, approximately 10 times higher than those from the LC–MS/MS method presented in Luche *et al.* (2019), which however used 400 mg of blubber. In the present study, we used Oasis PRiME polymeric sorbent SPE cartridges to provide a cleaner extract with less interferences. The hydrophilic and lipophilic groups attached to the copolymer sorbent allows high and reproducible recoveries of multiple steroid compounds. Our method uses the addition of the internal standard before sample processing to compensate for losses, sample-to-sample biological differences, and variability in interfering compounds, ensuring a reliable quantification of the compounds. The stable isotope-labelled internal standards for all eight steroids facilitate the validation study by using them to spike blubber homogenates to evaluate the required parameters. It is also much easier to determine the LODs of each steroid by spiking the blubber material with the labelled standards at a known concentration, which is not possible to evaluate when using pure steroid standard, especially when samples contain the analyte of interest (endogenous steroid hormones). By optimizing the HPLC gradient programme and choosing a Kinetex-F5 core-shell separation phase with higher backpressures that the system can handle, we obtain higher and narrower peaks, and a much better S/N compared to a fully porous column. This column with multiple interactive mechanisms has a very high degree of steric selectivity to separate structural isomers, such as 17-hydroxyprogesterone and 11-deoxycorticosterone. By using acetonitrile as mobile phase, the backpressure is reduced, the retention times are shorter, and the background noise is lower compared to methanol. A divert valve and needle wash is included in our method to prevent contamination and minimize MEs, in addition to the wash step from the gradient programme, to keep the column clean during each run. The sensitivity is also increased by using formic acid as an additive in the mobile phase, and the formation of the stable formate adduct into the ion source is facilitated. The ion source parameters were also optimized to enhance the sensitivity of all steroids, and a negative MRM acquisition mode was chosen for cortisone and cortisol to ensure much lower detection levels compared to the positive MRM acquisition mode. The injection volume was optimized to 20 μl to ensure good sensitivity of all steroids and optimal MEs that could increase by injecting a higher volume. A lower injection volume leads to less interference and lower background noise, with the sensitivity being affected at the same time.

The present LOD and LOQ for cortisol and cortisone were lower than for the other analysed hormones. By analysing cortisol and cortisone in negative mode using the formate-ion adducts, we achieved a very good signal-to-noise relationship, with a high sensitivity of peaks being monitored and very low background noise (Fig. 1). A higher background noise in positive mode leads to a lower signal-to-noise ratio, which means higher detection limits for the rest of steroids (Fig. 1). The lower observed detection frequency in 11-deoxycortisol, 11-deoxycorticosterone and 17-hydroxyprogesterone was similarly observed in other studies (Luche *et al.,* 2019; Boggs *et al.,* 2017), and can be due to a combination of higher LODs for these hormones and lower expression. One killer whale, the sub-adult female dubbed “Elida,” had quantified concentrations of every one of the eight steroid hormones, including 17-hydroxyprogesterone, which was <LOD in all other samples. This individual was found stranded with a thin blubber layer, and had high concentrations of persistent organic pollutants (Gundersen *et al.,* 2024) and the highest corticosteroids concentrations of all samples, indicating a long period of stress prior to death. 17-Hydroxyprogesterone, in addition to being a metabolite of progesterone, is an important intermediate in the synthesis of cortisol, and it is possible that the high relative concentrations of 17-hydroxyprogesterone, as well as 11-deoxycorticosterone and 11-deoxycortisol, are related to the extremely elevated concentrations of cortisol and cortisone in this individual compared to the other individuals.

Our method quantified the same concentrations of hormones in varying sample masses, with acceptable recovery and precision values, including in as little as 14-mg blubber in a sperm whale. We furthermore had the same detection rate between sample masses and illustrated that small blubber samples (50 mg) are sufficient for the simultaneous quantification of eight steroid hormones in these species, with strong indication that smaller sample sizes could also be sufficient, although further research and a larger sample size is needed for validation. This has important implications for further studies, as a smaller sample mass increases the likelihood of researchers conducting steroid hormone analyses, when the valuable blubber biopsy sample can also be used for other analyses. Moreover, by conducting multiple analyses on the same sample, complementary data can be obtained, which can further aid in interpreting steroid hormone concentrations, such as pollution levels, nutritional status and diet.

Hormone concentrations were within, or close to, the ranges previously reported in blubber of other whale species, which further supports the validity of our method. Mean cortisol concentrations in the stranded whale species from the present study ranged from 0.64 ng/g (harbour porpoise) to 4.3 ng/g (sperm whale), which is 1.2–10 times lower than mean cortisol concentrations in stranded humpback whales (5.2 ng/g; Luche *et al.,* 2019). Mean cortisol concentrations in the free-living killer whales (0.23 ng/g) and harvested minke whales (0.21 ng/g) were similar to the range found in free-living humpback whales (0.23–0.56 ng/g; Pallin *et al.,* 2022), and 10 times lower than free-living grey whales (mean, 2.8 ng/g; Wittmaack *et al.,* 2022). Cortisone concentrations (mean, 0.47–1.7 ng/g across all species) were similar to stressed bottlenose dolphins (mean, 1.7 ng/g; Galligan *et al.,* 2019) and stranded humpback whales (mean, 0.3–2.3 ng/g; Luche *et al.,* 2019). Our progesterone concentrations in minke whales (male, 2.1 ± 1.9 ng/g; pregnant female, 61 ± 34 ng/g) were within the same range as those found in the same population two decades ago using an immunoassay (male mean, 1.9 ± 0.27 ng/g; pregnant female mean, 130 ± 23 ng/g; Mansour *et al.,* 2002).

Furthermore, our results make biological sense in the context of what is known about how steroid hormone concentrations can vary in marine mammals due to age, sex and fatality type. Progesterone is the most widely used marker for diagnosing pregnancy in marine mammals in both blubber and serum (Mansour *et al.,* 2002; Robeck *et al.,* 2016; Pallin *et al.,* 2018; Hudson *et al.,* 2024), and we found elevated progesterone concentrations in the pregnant female minke whales (confirmed by presence of a foetus during harvesting). Testosterone has been used a biomarker for male sexual activity and maturity status in whales (Vu *et al.,* 2015; Cates *et al.,* 2019), and was higher in dominant, sexually mature male humpback whales (Mingramm *et al.,* 2020). We accordingly found the highest testosterone levels within each species in male individuals. Corticosteroid hormones are often lower in individuals that had a shorter duration of stress prior to death, due to a shorter time for cortisol to diffuse from blood to blubber (Schwacke and Wells, 2016; Champagne *et al.,* 2018), as observed in stranded vs by-caught short-beaked common dolphins (*Delphinus delphis*) (Kellar *et al.,* 2015), ice entrapped vs regularly harvested beluga whales (*Delphinapterus leucas*) (Trana *et al.,* 2016) and stranded vs free-living humpback whales (Mingramm *et al.,* 2020; Wittmaack *et al.,* 2022). In the present study, we observed a similar pattern, with low observed corticosteroid concentrations in minke whales, likely because minke whale harvesting in Norway involves little chase, and an instantaneous death in approximately 82% of cases (Øen, 2020). Similarly, corticosteroid concentrations in the free-living killer whales were four times lower than the stranded, likely due to the biopsy sampling involving only a small- and short-term stress response. In contrast, the higher corticosteroid concentrations in the stranded sperm and killer whales are likely due to a prolonged period of illness and/or starvation prior to death.

Our study is the first quantification of steroid hormones in killer whale, harbour porpoise and sperm whale blubber, and of a hormone other than progesterone in minke whale blubber. Our method is robust, sensitive and specific enough to detect eight steroid hormones simultaneously in 50 mg of whale blubber, with strong indications that lower sample masses could also be used. Application of our method can enable species comparisons and responses to various stressors, as well as indications of pregnancy and sexual maturity, to allow for effective conservation and management of these species.

## Supplementary Material

Web_Material_coag023

## Data Availability

The data underlying this article are available in the article and in its online supplementary material.
