# Supplementary material for: A sensitive liquid chromatography–tandem mass spectrometry analytical method of steroid hormones in small blubber samples from four whale species
Source: Conserv Physiol. 2026 Apr 13;14(1):coag023. doi: 10.1093/conphys/coag023 (PMC13076008; doi:10.1093/conphys/coag023)
Supplement: Web_Material_coag023 [file web_material_coag023.zip › Graphical abstract.pdf]

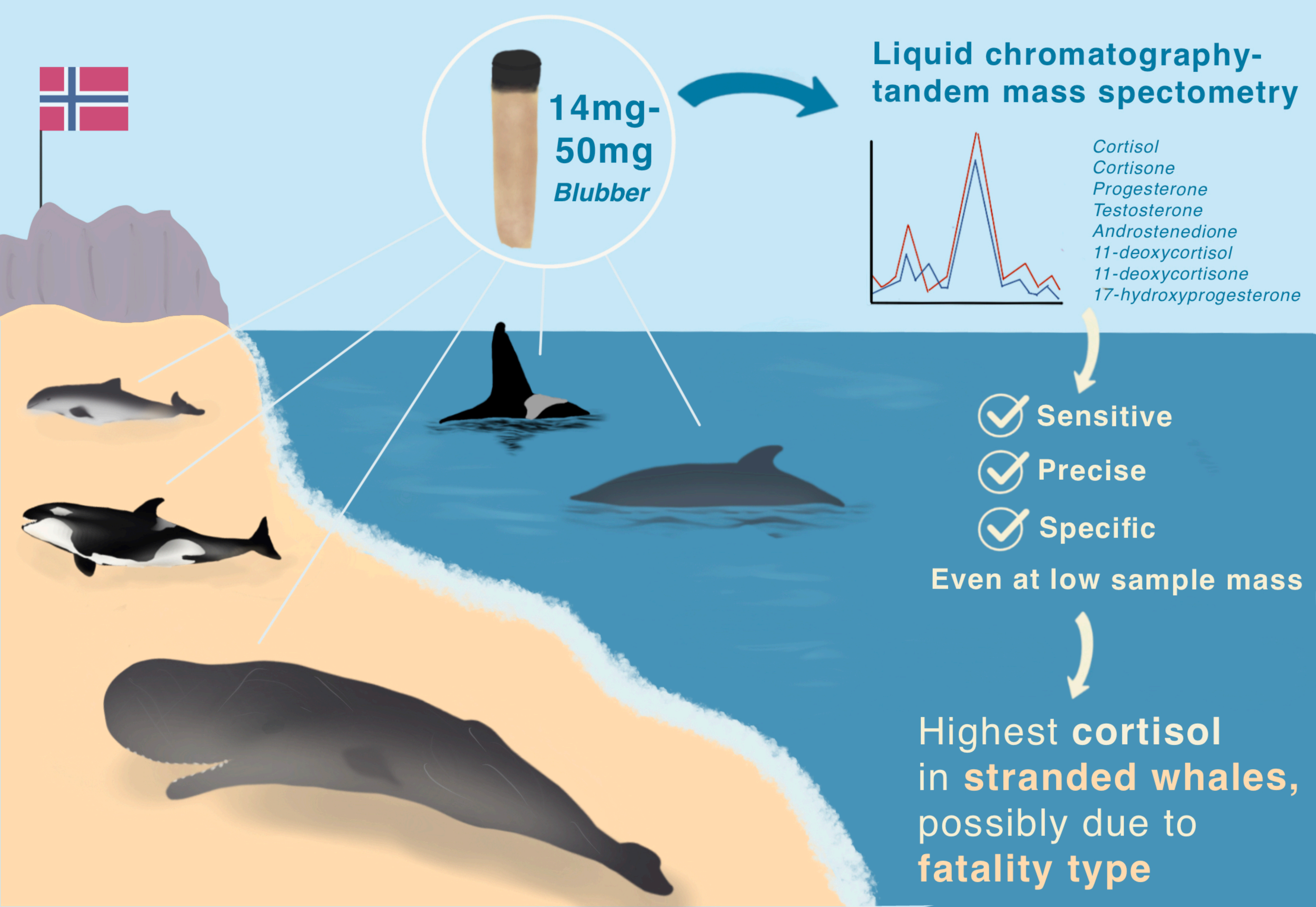
14mg-
50mg
Blubber
Liquid chromatography-
tandem mass spectometry
Cortisol
Cortisone
Progesterone
Testosterone
Androstenedione
11-deoxycortisol
11-deoxycortisone
17-hydroxyprogesterone
Sensitive
Precise
Specific
Even at low sample mass
Highest cortisol in stranded whales, possibly due to fatality type
