# Supplementary material for: A sensitive liquid chromatography–tandem mass spectrometry analytical method of steroid hormones in small blubber samples from four whale species
Source: Conserv Physiol. 2026 Apr 13;14(1):coag023. doi: 10.1093/conphys/coag023 (PMC13076008; doi:10.1093/conphys/coag023)
Supplement: Web_Material_coag023 [file web_material_coag023.zip › Supplementary_materials.pdf]

Supplementary Materials for:

A sensitive liquid chromatography-tandem mass spectrometry analytical method of steroid hormones in small blubber samples from four whale species

Daniela Dulgheriu[a, 1], Clare Andvik[b,1]*, Eve Jourdain[b,c], Katrine Borgå[b], Anders Ruus[b,e], Tore Haug[d], Richard Karoliussen[c], Jan Ludvig Lyche[a], Tor Einar Horsberg[a]*

[a] Department of Paraclinical sciences, Pharmacology/Toxicology Unit, Norwegian University of Life Sciences, 1433 Ås, Norway

[b] Department of Biosciences, University of Oslo, 0316 Oslo, Norway

[c] Norwegian Orca Survey, 8480 Andenes, Norway

[d] Institute of Marine Research, Fram Centre, Pb 6606 Stakkevollan, NO–9296 Tromsø, Norway

[e] Norwegian Institute for Water Research, 32821 Oslo, Norway

[1]Contributed equally, shared first authorship

*Correspondence to: Clare Andvik, clarem@uio.no and Daniela Dulgheriu daniela.dulgheriu@nmbu.no

Summary: 18 pages, 5 figures, 1 text, 8 tables

Table of Contents

Supplementary Figure S1: Map of Scandinavia, the Barents Sea and Svalbard showing where each of the 26 marine mammals were sampled....3

Supplementary Table S1: Blubber steroid hormone levels (ng/g) in a stranded killer whale (ID Oo3) homogenised by 1) Knife Mill Grindomix 200 machine and dry ice and 2) pestle and mortar with liquid nitrogen....4

Supplementary Text 1: Method parameters used for the simultaneous analysis of eight steroid hormones in whale blubber....5

Supplementary Table S2: Assay performance parameters: intra-assay accuracy and precision for spiked blubber whale samples for eight steroid hormones....6

Supplementary Table S3: Assay performance parameters: inter-assay accuracy and precision for spiked blubber whale samples for eight steroid hormones....7

Supplementary Table S4: Matrix effect and extraction recovery for eight steroid hormones using pre-spiked, post-spiked whale-blubber "blanks" and pure standard mixture...8

Supplementary Figure S2: Extracted ion chromatograms of endogenous steroid hormones in A) a 20 mg sample from sperm whale (Physeter macrocephalus) B) a 25 mg blubber samples from killer whale (Orcinus orca) C) Extracted ion chromatograms of cortisol and cortisol-d4 internal standard in 14 g tested sperm whale blubber sample and D) Extracted ion chromatograms of cortisol and cortisol-d4 internal standard in 50 mg tested sperm whale blubber sample...9

Supplementary Table S5: Summary statistics for five steroid hormones (ng/g) quantified in four species of whale from northern Norway, including both stranded and free-ranging killer whales...13

Supplementary Table S6: Point estimates, standard error, degrees of freedom (df), lower and upper confidence intervals (CI), t-ratio and p-values for pairwise species computed from the linear regression log10cortisol ~ Species...14

Supplementary Table S7: Point estimates, standard error, degrees of freedom (df), lower and upper confidence intervals (CI), t-ratio and p-values for pairwise species computed from the linear regression log10cortisone ~ Species....15

Supplementary Figure S3: Progesterone levels (ng/g) in four species of whales sampled in Norway, divided by sex....14

Supplementary Figure S4: Testosterone levels (ng/g) in four species of whales sampled in Norway, divided by sex....17

Supplementary Figure S5: Androstenedione levels (ng/g) in four species of whales sampled in Norway, divided by sex....15

Supplementary Table S8: Results from the mixed effect models fitted to investigate the effect of Age/Sex and Lipid % on blubber steroid hormone levels in four species of whale sampled from the Norwegian coast and Barents Sea 2016–202....19

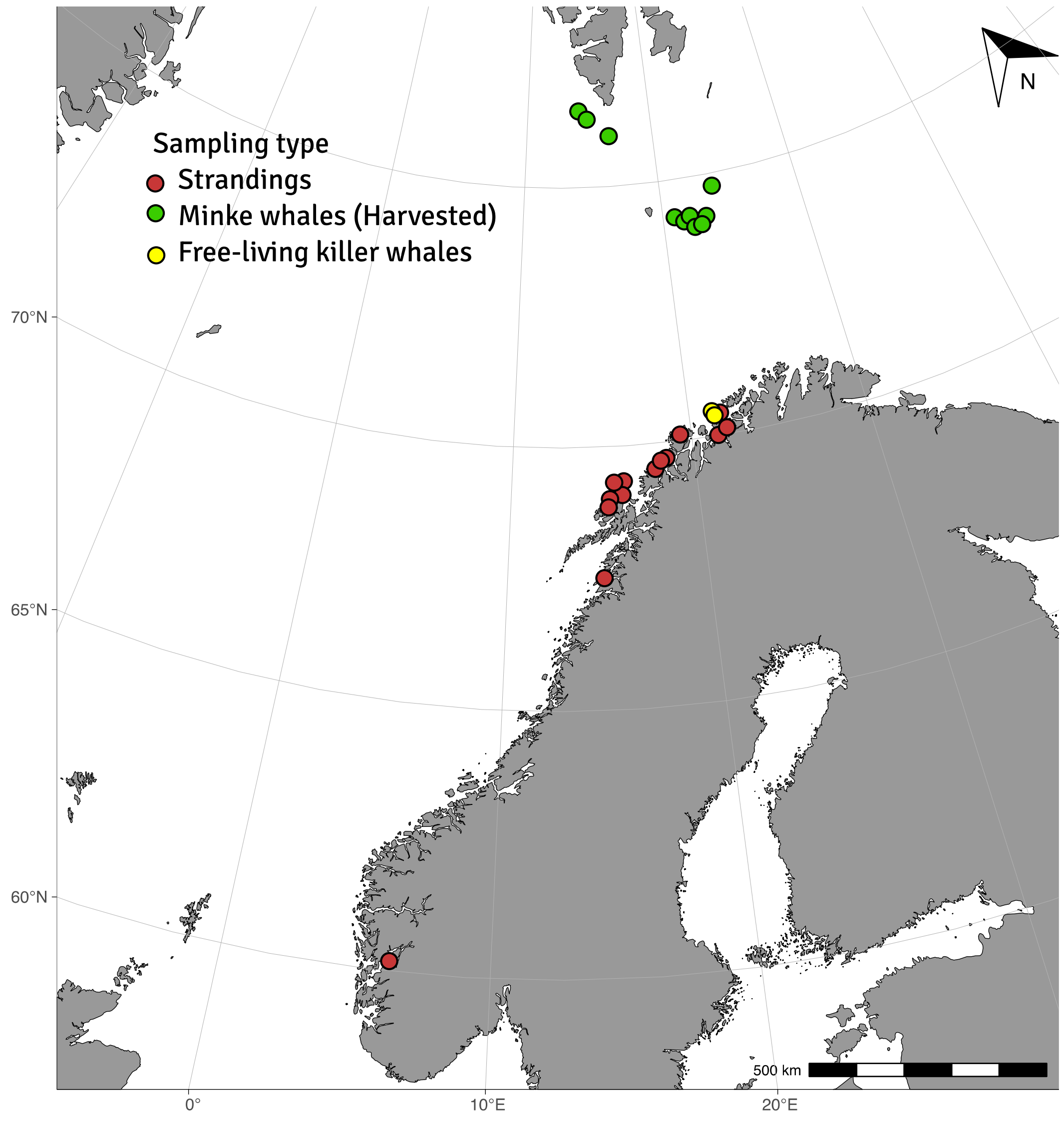

Supplementary Figure S1: Map of Scandinavia, the Barents Sea and Svalbard showing where each of the 26 marine mammals were sampled. Green dots represent the 10 common minke whales obtained in 2019 from the annual harvest in the Barents Sea, red dots the 14 marine mammals that stranded along the Norwegian coastline from 2016–2021, and yellow dots the two free-ranging killer whales samples in 2021.

Supplementary Table S1: Blubber steroid hormone levels (ng/g) in a stranded killer whale (ID Oo3) homogenised by 1) Knife Mill Grindomix 200 machine and dry ice and 2) pestle and mortar with liquid nitrogen. A linear regression was fitted for each hormone: log10hormone ~ Homogenisation technique. Standard deviation across all values, and the back-transformed standard error, effect size, confidence intervals and p-value from the linear model are listed. An effect size close to 1 indicates a negligible effect of homogenisation method on hormone levels.

**Whale ID**	**Weight (mg)**	**Homogenisation technique**	**Cortisol ng/g**	**Cortisone ng/g**	**Testosterone ng/g**	**Prog-esterone ng/g**	**Andro-stenedione ng/g**
Oo3	50	Lyser machine	2.0	1.1	< 0.50	68	4.0
Oo3	50	Pestle & mortar	1.9	0.93	< 0.50	69	4.0
Oo3	25	Lyser machine	2.1	1.1	< 0.50	68	4.1
Oo3	26	Pestle & mortar	1.9	0.95	< 0.50	68	4.1
Standard deviation			0.080	0.080	NA	0.19	0.050
Standard error			0.023	0.016	NA	0.0030	0.017
Effect size			0.93	0.86	NA	1.0	1.0
Confidence intervals			0.84–1.0	0.80–0.12	NA	0.98–1.00	0.94–1.1
p-value			0.12	0.16	NA	0.54	0.69

Supplementary Text 1: Method parameters used for the simultaneous analysis of eight steroid hormones in whale blubber.

Liquid chromatography method

Agilent 1100 setup binary pump and autosampler
Mobile phase A: 0.1% HCOOH in H_2O
Mobile phase B: : 0.1% HCOOH in Acetontrile
Column: Phenomenex Kinetex – F5, 100 x 2.1 mm i.d. 2.6µm
Injection volume: 20 µL
Flow rate: 0.25 mL/min
Autosampler temperature: 5 °C
Column temperature: 35 °C
Needle wash: 0.2%HCOOH in (25% IPA: 25% MeOH: 25% AcN: 25% H_2O

Gradient:	Time	%B
	0.00	15
	2.00	15
	9.60	85
	10.0	90
	10.5	95
	11.0	95
	12.0	98
	12.2	100
	15.0	100
	15.1	15
	20.0	15

MS/MS-Method

API 4000 triple-quadruple mass spectrometer

Scan type: MRM
Ion mode: ESI neg/ ESI pos
Ion spray voltage: -4400V/5500V
Q1/Q3 resolution: Unit
Curtain gas (N_2): 20 psi
Nebuliser gas (GSI)(N_2): 50 psi
Turbo gas (GS2)(N_2)

Supplementary Table S2: Assay performance parameters: intra-assay accuracy and precision for spiked blubber whale samples for eight steroid hormones.

Analyte	Concentration(ng/g)	Intra-assay (N=6)	
		Accuracy (%)	Precision (RSD%)
Cortisol	5.0	100	8.4
Cortisone	5.0	96	5.1
Androstenedione	5.0	100	2.2
Testosterone	5.0	99	4.5
11-deoxycorticosterone	5.0	97	2.4
17-hydroxyprogesterone	5.0	99	4.9
Progesterone	5.0	100	5.8
11-deoxycortisol	5.0	100	8.3

Supplementary Table S3: Assay performance parameters: inter-assay accuracy and precision for spiked blubber whale samples for eight steroid hormones.

Analyte	Concentration(ng/g)	N	Inter-assay	
			Accuracy (%)	Precision (RSD%)
Cortisol	0.22	4	101	3.2
	0.75	5	100	1.7
	1.3	4	93	1.2
	4.3	4	100	2.3
Cortisone	0.27	4	90	5.8
	0.77	5	98	5.1
	1.8	3	93	2.8
	4.3	4	100	2.8
Androstenedione	1.4	4	103	2.8
	1.9	4	110	5.1
	2.4	5	110	1.5
	5.4	3	110	2.8
Testosterone	0.3	4	112	2.8
	0.8	4	100	4.9
	1.3	5	100	5.6
	4.3	5	100	7.9
11-deoxycorticosterone	0.5	4	110	11
	1.0	5	100	7.5
	4.0	5	110	3.1
17-hydroxyprogesterone	0.50	4	110	8.0
	1.0	5	110	6.0
	4.0	5	110	3.3
Progesterone	0.70	4	91	9.0
	1.2	5	99	4.8
	4.2	3	100	2.9
11-deoxycortisol	0.50	4	100	5.6
	1.0	5	110	4.1
	4.0	5	100	5.56

Supplementary Table S4: Matrix effect and extraction recovery for eight steroid hormones using pre-spiked, post-spiked whale-blubber "blanks" and pure standard mixture

**Compound**	**Extraction efficiency (%)**	**Matrix effect (ME) (%)**
Cortisol	98	65
Cortisone	88	71
Androstenedione	117	65
Testosterone	109	79
11-deoxycorticosterone	116	81
17-hydroxyprogesterone	95	90
Progesterone	101	86
11-deoxycortisol	113	91

Supplementary Figure S2: Extracted ion chromatograms of endogenous steroid hormones in A) a 20 mg sample from sperm whale (*Physeter macrocephalus)* B) a 25 mg blubber samples from killer whale (*Orcinus orca)* C) Extracted ion chromatograms of cortisol and cortisol-d4 internal standard in 14 g tested sperm whale blubber sample and D) Extracted ion chromatograms of cortisol and cortisol-d4 internal standard in 50 mg tested sperm whale blubber sample

A)

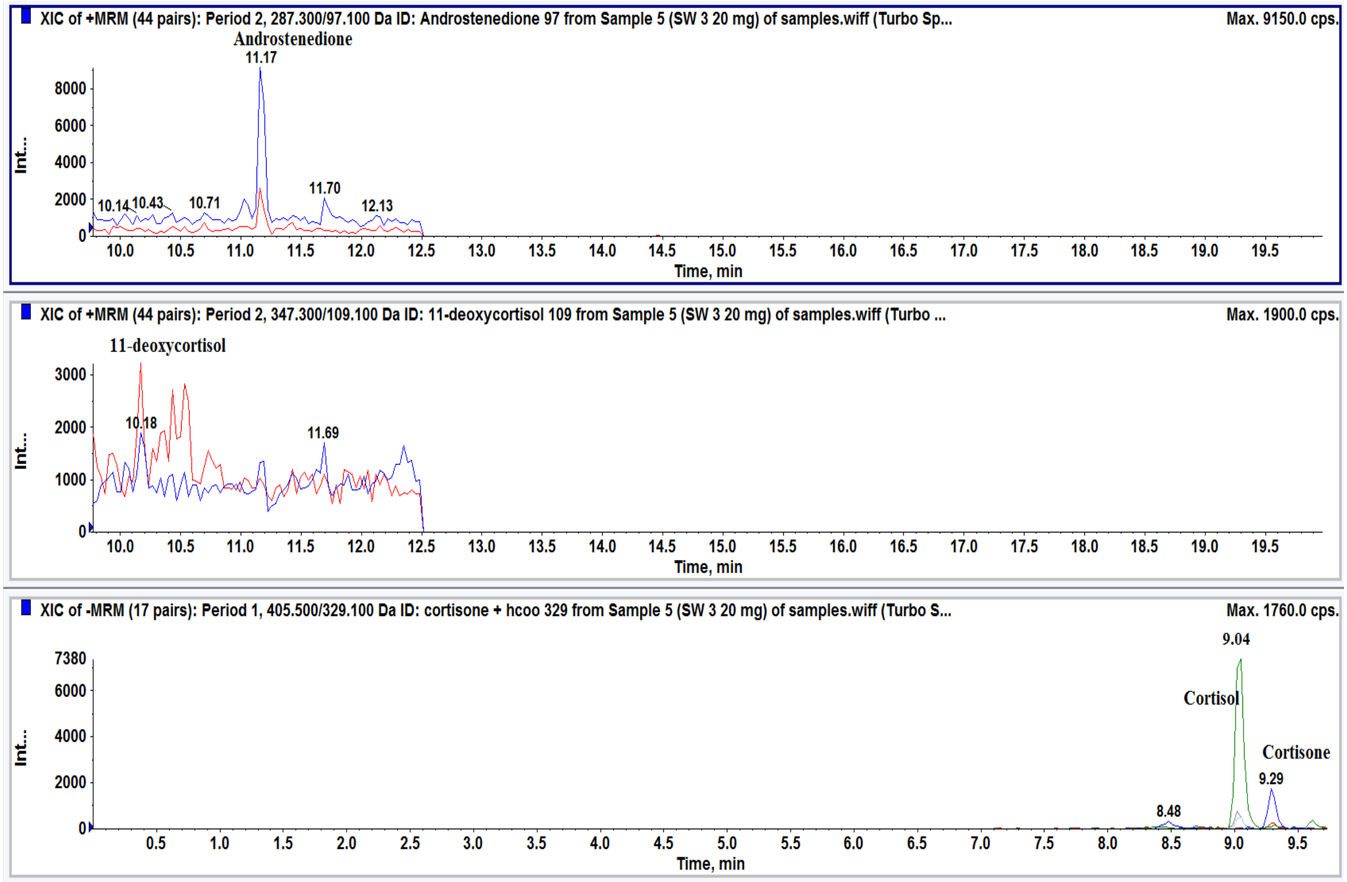

B)

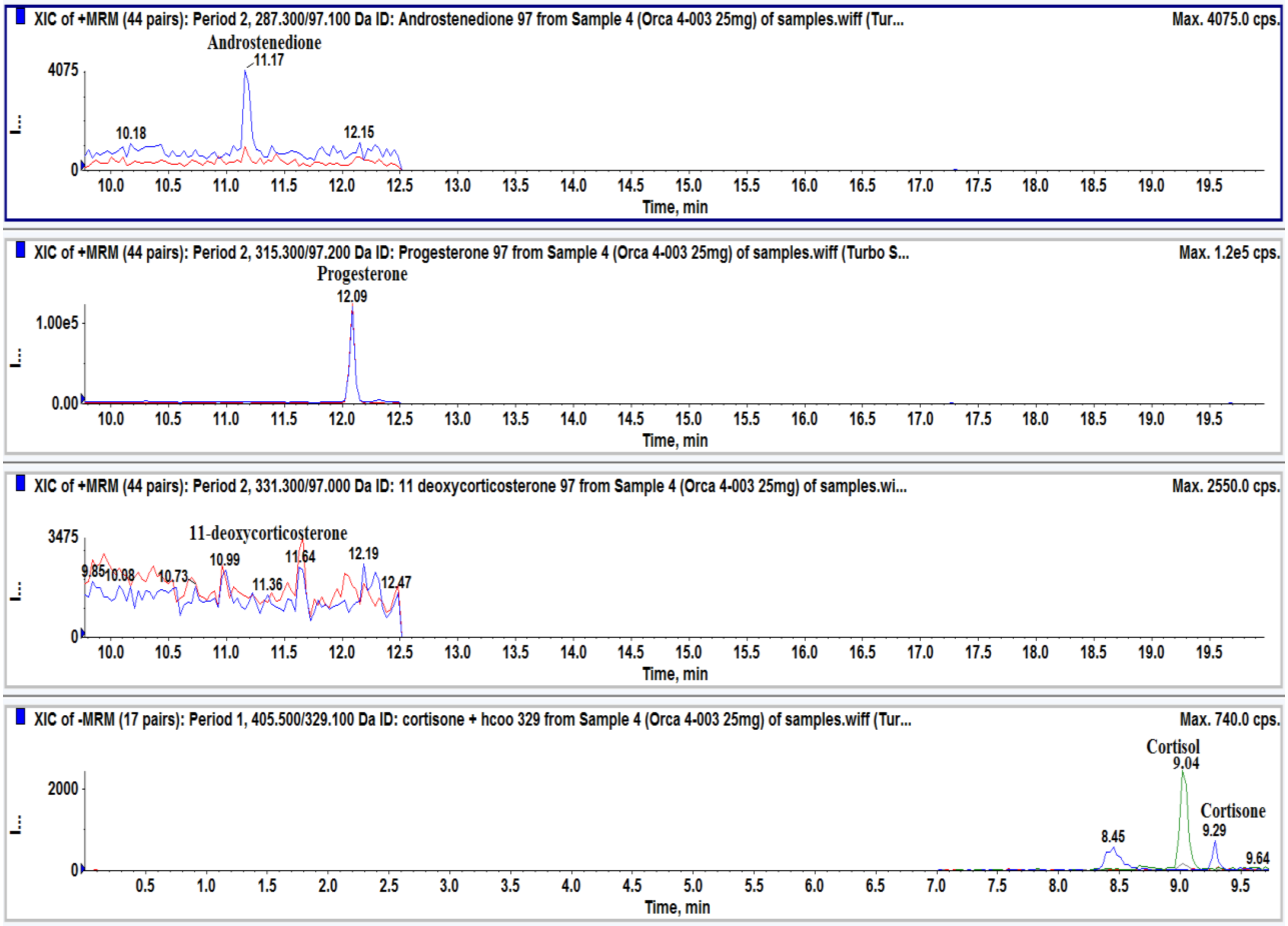
XIC of +MRM (44 pairs): Period 2, 287.300/97.100 Da ID: Androstenedione 97 from Sample 4 (Orca 4-003 25mg) of samples.wiff (Tur...
Max. 4075.0 cps.
Androstenedione
11.17
10.18
12.15
Time, min
XIC of +MRM (44 pairs): Period 2, 315.300/97.200 Da ID: Progesterone 97 from Sample 4 (Orca 4-003 25mg) of samples.wiff (Turbo S...
Max. 1.2e5 cps.
Progesterone
12.09
Time, min
XIC of +MRM (44 pairs): Period 2, 331.300/97.000 Da ID: 11 deoxycorticosterone 97 from Sample 4 (Orca 4-003 25mg) of samples.wi...
Max. 2550.0 cps.
11-deoxycorticosterone
Time, min
XIC of -MRM (17 pairs): Period 1, 405.500/329.100 Da ID: cortisone + hcoo 329 from Sample 4 (Orca 4-003 25mg) of samples.wiff (Tur...
Max. 740.0 cps.
Cortisol
9.04
Cortisone
9.29
8.45
9.64
Time, min

C)

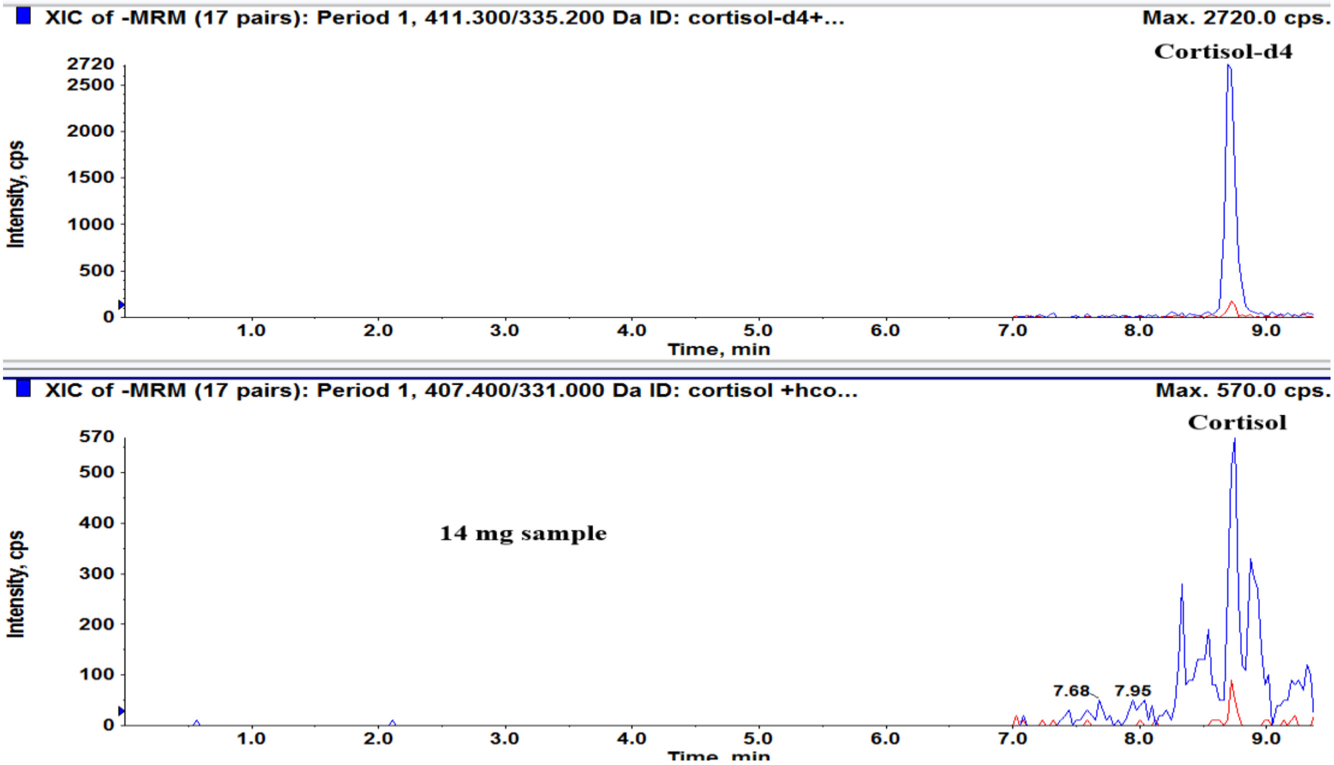

XIC of -MRM (17 pairs): Period 1, 411.300/335.200 Da ID: cortisol-d4+...
Max. 2720.0 cps.
Cortisol-d4
Intensity, cps
2720
2500
2000
1500
1000
500
0
1.0
2.0
3.0
4.0
5.0
6.0
7.0
8.0
9.0
Time, min
XIC of -MRM (17 pairs): Period 1, 407.400/331.000 Da ID: cortisol +hco...
Max. 570.0 cps.
Cortisol
14 mg sample
Intensity, cps
570
500
400
300
200
100
0
7.68
7.95
1.0
2.0
3.0
4.0
5.0
6.0
7.0
8.0
9.0

D)

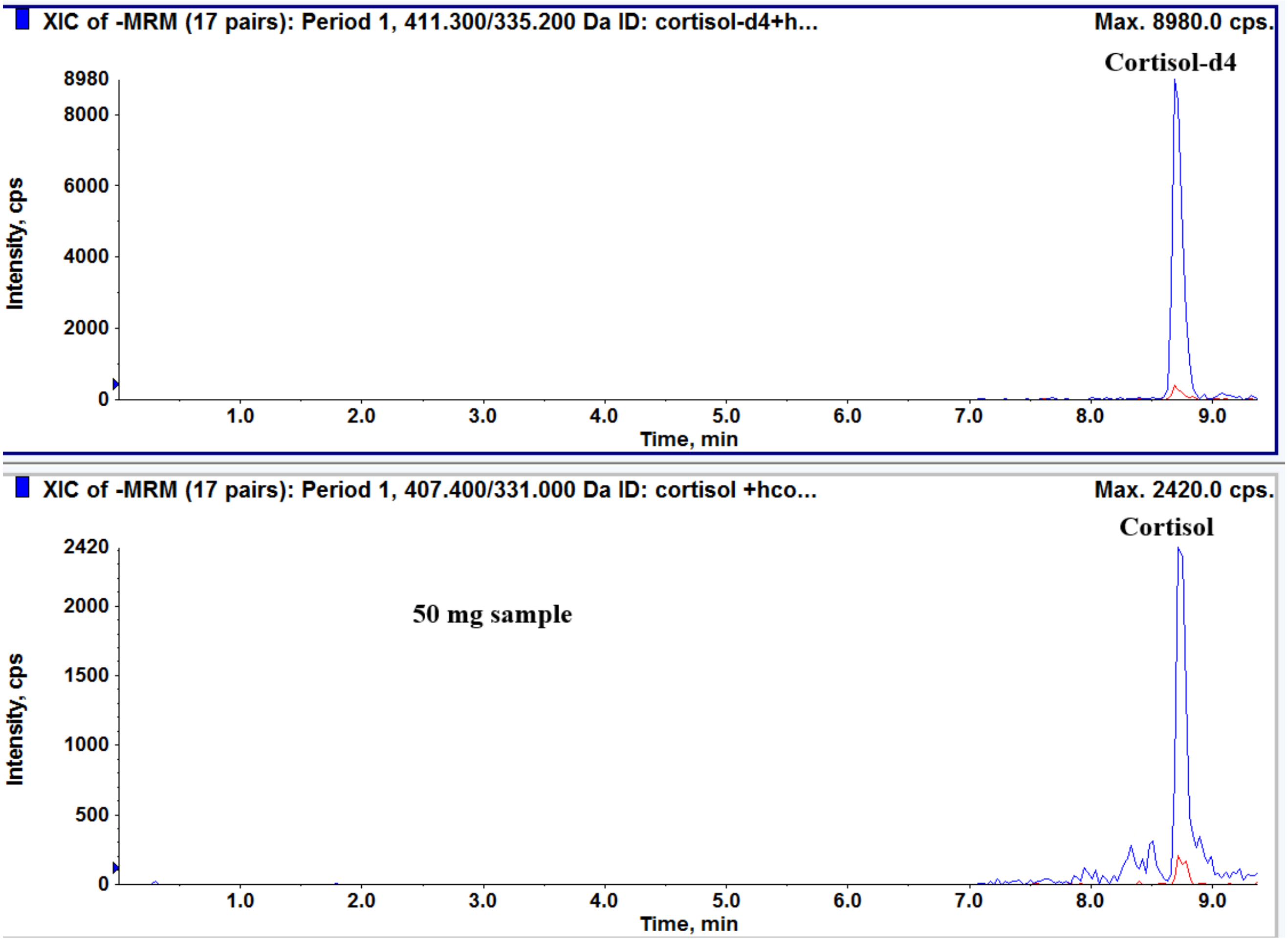
XIC of -MRM (17 pairs): Period 1, 411.300/335.200 Da ID: cortisol-d4+h...
Max. 8980.0 cps.
Cortisol-d4
8980
8000
6000
4000
2000
0
Intensity, cps
1.0
2.0
3.0
4.0
5.0
6.0
7.0
8.0
9.0
Time, min
XIC of -MRM (17 pairs): Period 1, 407.400/331.000 Da ID: cortisol +hco...
Max. 2420.0 cps.
Cortisol
50 mg sample
2420
2000
1500
1000
500
0
Intensity, cps
1.0
2.0
3.0
4.0
5.0
6.0
7.0
8.0
9.0
Time, min

Supplementary Table S5: Summary statistics for five steroid hormones (ng/g) quantified in four species of whale from northern Norway, including both stranded and free-ranging killer whales. Mean ± SD, median and range are provided for each Age/Sex category, in addition to each species as a whole. The values for the stranded killer whales include subadult female with ID Oo11 "Elida", which is treated as an outlier in the main text.

Species	Age & Sex	*n*	**Cortisol (ng/g)**		**Cortisone (ng/g)**		**Testosterone (ng/g)**		**Progesterone (ng/g)**		**Androstenedione (ng/g)**	
			Mean ± SD	Median (Min–Max)	Mean ± SD	Median (Min–Max)	Mean ± SD	Median (Min–Max)	Mean ± SD	Median (Min–Max)	Mean ± SD	Median (Min–Max)
Minke whale (Harvested) 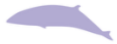	Adult female	5	0.21 ± 0.060	0.19 (0.16–0.29)	0.34 ± 0.11	0.29 (0.28–0.52)	0.25 ± 0.13	0.24 (0.10–0.42)	61 ± 33	62 (20–100)	2.1 ± 0.95	2.1 (1.0–3.3)
	Adult male	4	0.17 ± 0.050	0.19 (0.12–0.21)	0.60 ± 0.46	0.42 (0.28–1.4)	1.2 ± 0.83	1.0 (0.25–2.5)	2.1 ± 1.9	1.3 (0.62–5.2)	7.4 ± 8.9	4.4 (2.3–23)
	Subadult female	1	0.55	0.55	0.35	0.35	1.2	1.2	1.4	1.4	12	12
	**All**	10	0.22 ± 0.13	0.2 (0.12–0.55)	0.47 ± 0.34	0.35 (0.28–1.4)	0.80 ± 0.73	0.59 (0.10–2.5)	26 ± 36	3.7 (0.62–100)	5.8 ± 6.9	2.9 (1.0–23)
Killer whale (Free-living)	Adult male	2	0.23 ± 0.12	0.23 (0.14–0.31)	0.17 ± 0.010	0.17 (0.16–0.18)	3.3 ± 1.9	3.3 (2-0–4.6)	0.40 ± 0.44	0.40 (0.090–0.72)	1.3 ± 0.34	1.3 (1.0–1.5)
Killer whale (stranded) 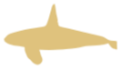	Adult female	3	1.7 ± 0.91	2.0 (0.72–2.5)	1.3 ± 0.35	1.2 (1.1–1.7)	0.42 ± 0.36	0.22 (0.20–0.83)	25 ± 37	3.5 (2.9–68)	2.4 ± 1.5	2.2 (1.0–4-0)
	Adult male	2	0.71 ± 0.69	0.71 (0.22–1.2)	0.64 ± 0.52	0.64 (0.27–1.0)	1.0 ± 1.0	1.0 (0.30–1.8)	0.68 ± 0.80	0.68 (0.11–1.2)	1.3 ± 0.17	1.25 (1.1–1.4)
	Subadult female	1	29	29	4.1	4.1	0.39	0.39	2.5	2.5	0.87	0.87
	Subadult male	1	0.11	0.11	0.36	0.36	0.070	0.070	0.27	0.27	2.9	2.9
	**All**	7	5.1 ± 11	1.2 (0.11–29)	1.4 ± 1.3	1.1 (0.27–4.1)	0.54 ± 0.59	0.30 (0.070–1.8)	11 ± 25	2.5 (0.11–68)	1.9 ± 1.2	1.4 (0.87–4.0)
Harbour porpoise (Stranded) 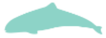	Unknown	3	0.64 ± 0.38	0.64 (0.25–1.0)	0.62 ± 0.17	0.71 (0.42–0.72)	0.58 ± 0.11	0.62 (0.46–0.66)	0.17 ± 0.12	0.17 (0.040–0.28)	3.7 ± 2.2	3.4 (1.6–6.0)
Sperm whale (Stranded) 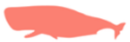	Adult female	1	2.9	2.9	1.1	1.1	0.78	0.78	1.5	1.5	2.3	2.3
	Adult male	2	4.0 ± 1.1	4.0 (3.2–4.7)	1.9 ± 0.94	1.9 (1.2–2.6)	1.3 ± 0.66	1.3 (0.84–1.8)	0.14 ± 0.060	0.14 (0.090–0.18)	4.0 ± 2.1	4.0 (2-6–5.5)
	Subadult male	1	6.3	6.3	1.8	1.8	1.6	1.6	0.66	0.66	3.1	3.1
	**All**	4	4.3 ± 1.6	4.0 (2.9–6.3)	1.7 ± 0.66	1.5 (1.1–2.6)	1.2 ± 0.51	1.2 (0.78–1.8)	0.61 ± 0.64	0.42 (0.090–1.5)	3.4 ± 1.5	2.8 (2.3–5.5)

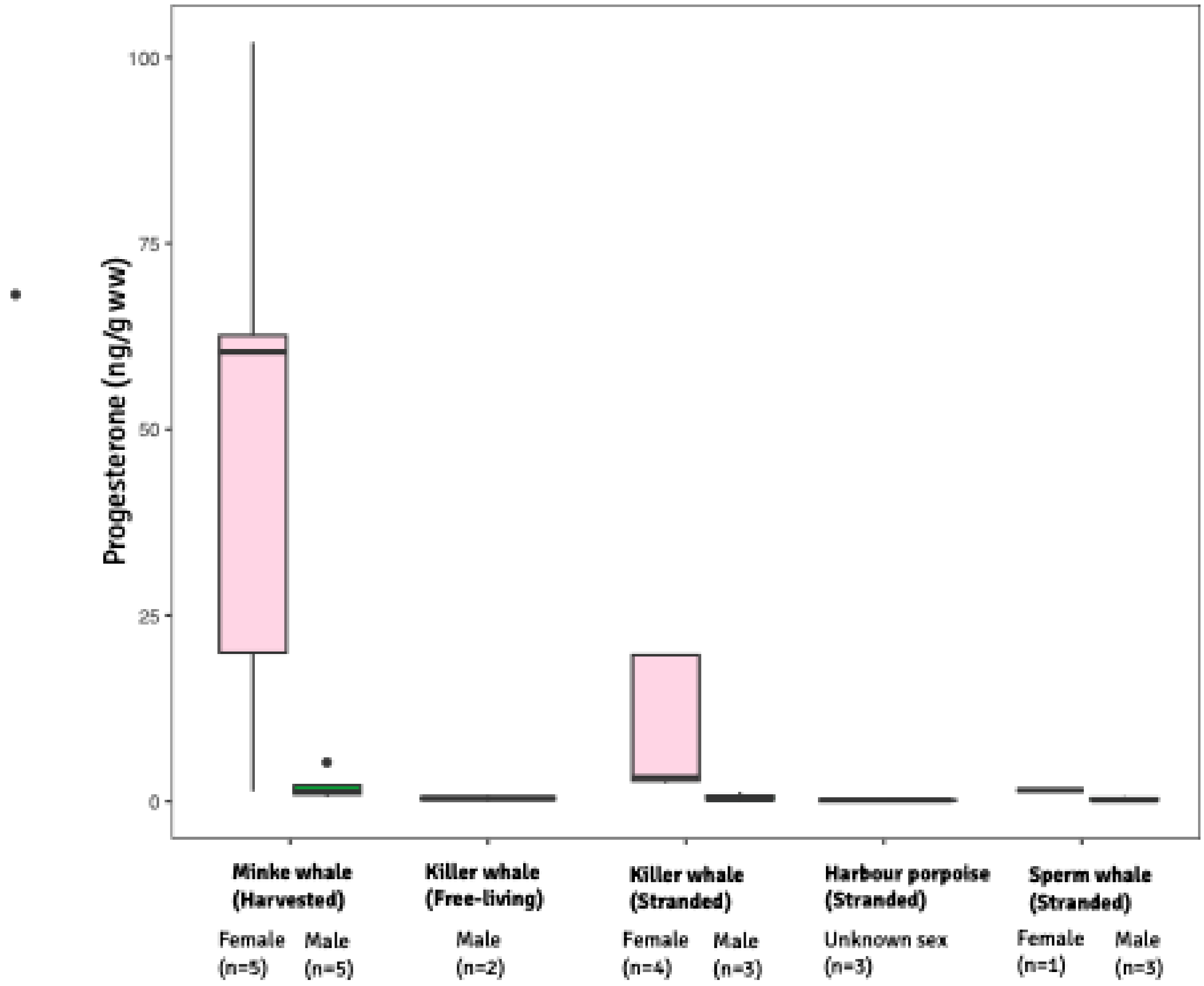

Supplementary Figure S3: Progesterone levels (ng/g) in four species of whales sampled in Norway, divided by sex.

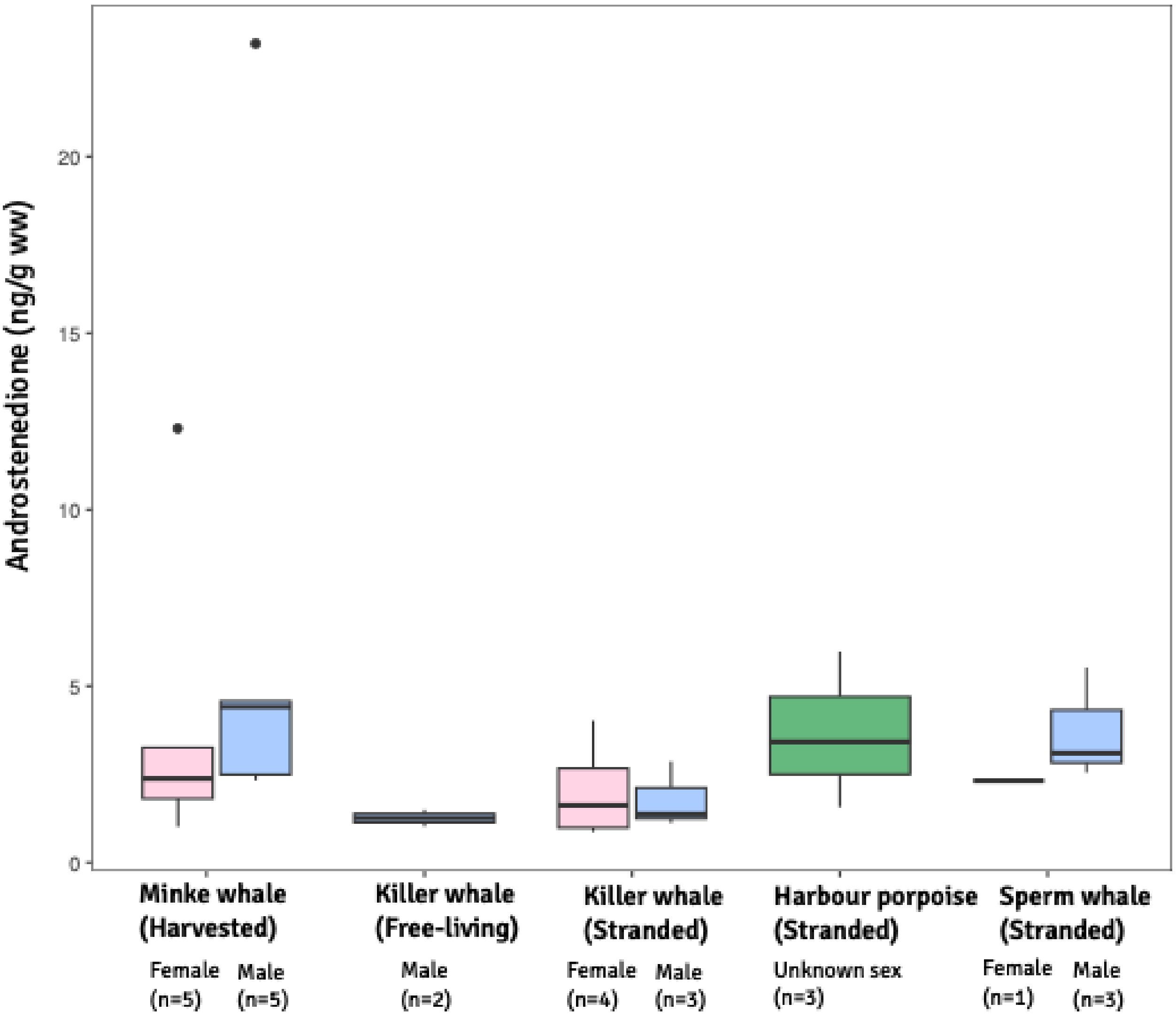

Supplementary Figure S4: Androstenedione levels (ng/g) in four species of whales sampled in Norway, divided by sex.

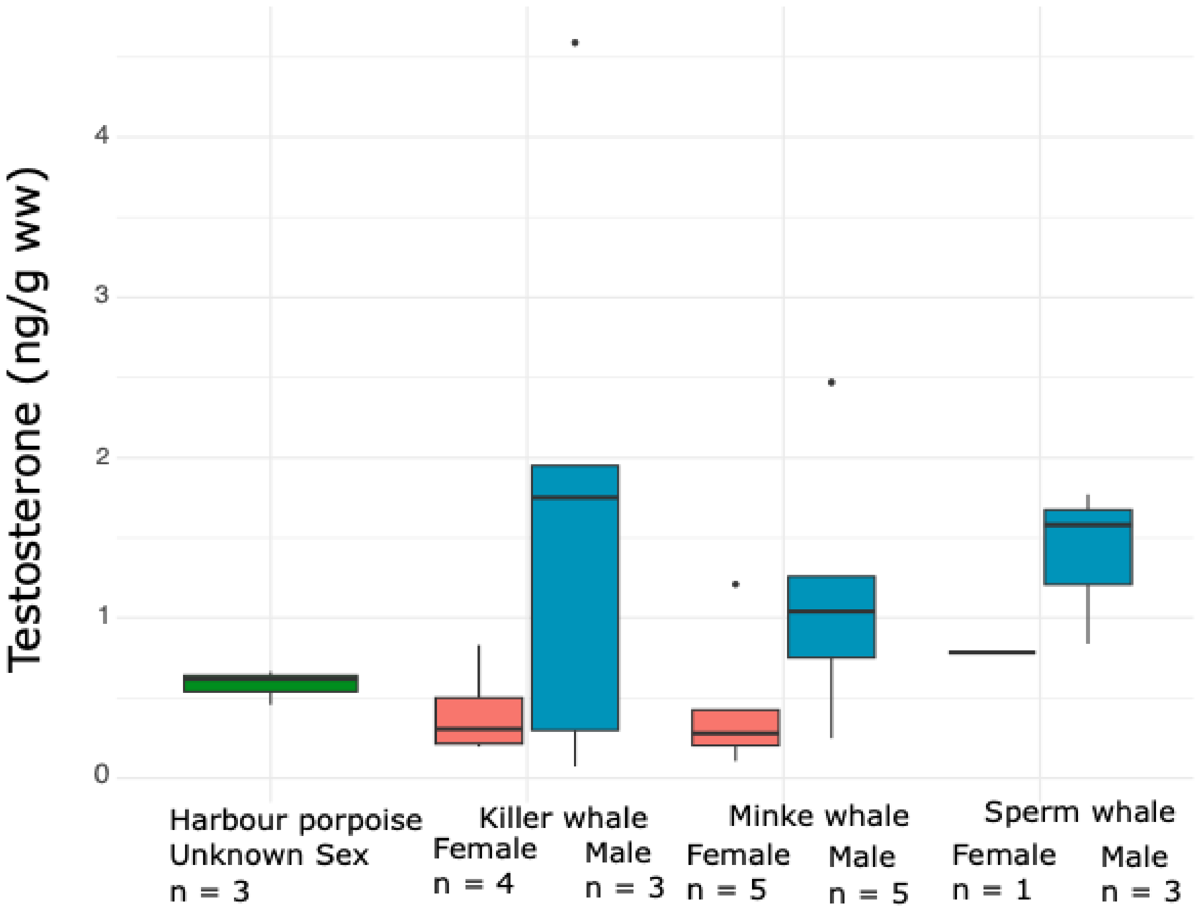

Supplementary Figure S5: Testosterone levels (ng/g) in four species of whales sampled in Norway, divided by sex
